# A critical review of graphics for subgroup analyses in clinical trials

**DOI:** 10.1002/pst.2012

**Published:** 2020-03-25

**Authors:** Nicolás M. Ballarini, Yi‐Da Chiu, Franz König, Martin Posch, Thomas Jaki

**Affiliations:** ^1^ Center for Medical Statistics, Informatics, and Intelligent Systems Medical University of Vienna Vienna Austria; ^2^ Royal Papworth Hospital NHS Foundation Trust London UK; ^3^ MRC Biostatistics Unit University of Cambridge School of Clinical Medicine Cambridge UK; ^4^ Medical and Pharmaceutical Statistics Research Unit, Department of Mathematics and Statistics Lancaster University Lancaster UK

**Keywords:** contour plot, data visualisation, exploratory data analysis, forest plot, Galbraith plot, STEPP, treatment effect heterogeneity, UpSet plot

## Abstract

Subgroup analyses are a routine part of clinical trials to investigate whether treatment effects are homogeneous across the study population. Graphical approaches play a key role in subgroup analyses to visualise effect sizes of subgroups, to aid the identification of groups that respond differentially, and to communicate the results to a wider audience. Many existing approaches do not capture the core information and are prone to lead to a misinterpretation of the subgroup effects. In this work, we critically appraise existing visualisation techniques, propose useful extensions to increase their utility and attempt to develop an effective visualisation approach. We focus on forest plots, UpSet plots, Galbraith plots, subpopulation treatment effect pattern plot, and contour plots, and comment on other approaches whose utility is more limited. We illustrate the methods using data from a prostate cancer study.

## 1 INTRODUCTION

Investigating target populations that potentially benefit from an innovative intervention is essential in clinical trials. Even if efficacy is established in the overall population, a complete benefit/risk assessment of subgroups should be undertaken before deciding whether the treatment is administered to the whole population or targeted to specific subgroups.^1^ Such investigations pose numerous challenges such as recruiting patients with diverse baseline characteristics, which may create a large number of subgroups. The presence of promising results in subgroup analyses can be attributed to small sample sizes or to the fact that many potential subgroups are explored, which affects the credibility of the findings.

Subgroup analyses might be prospective or post‐hoc in different settings of clinical trials. Their primary purpose could be to establish efficacy claims, subgroup discovery and/or consistency assessments across subgroups. Many researchers have proposed novel analysis approaches and trial designs for different types of subgroup analysis.^2^, ^3^, ^4^ Subgroups have further received extensive attention in recent clinical research for the development of stratified medicine.

Visualisation techniques, when properly used, are powerful tools. It is argued that graphics allow a more direct interpretation of results than tables.^5^ There is extensive literature on principles for good graphics in general^6^, ^7^, ^8^, ^9^, ^10^, ^11^, ^12^ particularly in visualisation of healthcare data.^13^, ^14^, ^15^ It is also true that good graphics require careful crafting^16^ and there is scope to improve when it comes to figures found in clinical trial reports.^17^, ^18^

Graphical approaches are routinely employed in subgroup analysis, typically for describing treatment effect sizes of subgroups. Such visualisations encapsulate subgroup information and boost the clinical decision‐making process. However, current literature does not adequately provide solutions to producing effective graphics in subgroup analyses. Existing approaches still have inherent drawbacks and their use may lead to misinterpretations of subgroup effect sizes.^2^

In this article, we critically evaluate and refine effective visualisation approaches for subgroup analysis. Our considerations apply mainly to exploratory settings. Some of these visualisations have previously been proposed for subgroup analysis and were refined in this work. There are existing alternative techniques primarily developed for other applications which we have applied and/or extended to provide visual insight of subgroup information.

The remainder of the article is structured as follows. In Section 2 we describe: the framework for assessment, the dataset we use for illustration, and the graphical approaches for displaying subgroup information. We focus on graphics that allow a direct comparison of subgroup treatment effects. We summarise the findings in the case study and the assessments and features of all graphical approaches in Section 3. Section 4 provides a conclusion with final remarks.

## 2 GRAPHICAL APPROACHES TO SUBGROUP PROBLEMS

### 2.1 Framework to assess the properties of the graphical displays

It is fundamental that graphics in subgroup analysis display treatment effects for the subgroups under considerations. There are several other desirable characteristics for graphical approaches as initial subgroup analysis tools. Displaying sample sizes and uncertainty measures underpins the credibility of promising and adverse findings within subgroups. While many subgroup analysis techniques consider subgroups that are defined based on each baseline factor separately (univariate subgroups), it is also important to reveal information on those defined based on multiple factors (multivariate subgroups). For example, instead of looking at the subgroups defined by gender (male/female) and bone metastasis (yes/no) separately, it may be of interest to look at the intersection of the marginal subgroups: male with bone metastasis, male without bone metastasis, female with bone metastasis, and female without bone metastasis. These characteristics can certainly constitute sensible criteria for assessment. Our framework to assess the properties of the graphical displays consists of the criteria outlined in Table 1.

**Table 1:** Criteria to assess the properties of the graphical displays

Criteria	Label	Description
C1	Effect size	Displays effect sizes for subgroups
C2	Uncertainty	Provides confidence intervals or standard errors of the treatment effect estimates
C3	Sample size	Exhibits subgroup sample sizes
C4	Intersections	Shows effect sizes for multivariate subgroups
C5	Many covariates	Applicable to a large number of subgroup‐defining covariates

Each graphical approach is judged according to whether it meets the criteria set out. Even if a criterion is met, the information may be represented or encoded differently. For example, some graphics show the treatment effects in the subgroups using a colour scale while others represent them with the position of a point along a common scale. We discuss different levels of information in each of the graphics.

### 2.2 Case study: The prostate cancer dataset

To illustrate the different graphical approaches, we use data from a prostate carcinoma clinical trial^19^ which is available on the web^20^ and has previously been used to demonstrate subgroup selection methods.^21^ The trial included 506 subjects that were randomised to either a placebo group or one of three dose levels of diethylstilbestrol. In line with previous work, we combine the placebo and the lowest dose level of diethylstilbestrol to give the control arm, and the higher doses to give the experimental arm. Only 475 subjects with complete data are used in our illustration. We aim to describe the estimates for treatment effect across the different subgroups of patients. To illustrate the graphics, we consider six pre‐treatment covariates, four of which are binary and two continuous: *existence of bone metastasis* (*bm*: 0, no; 1, yes), *disease stage* (3 or 4), *performance rating* (*pf*: 0, normal; 1, limitation of activity), *history of cardiovascular events* (*hx* 0, no; 1, yes), *age*, and *weight index* (*wt*: weight in kg − height in cm + 200). The considered endpoint in this analysis is death from all causes combined, and the log‐hazard ratio for treatment vs control is used as the treatment effect measure.

### 2.3 Visualisation methods

In this subsection, we present the graphical approaches that are best suited for subgroup analysis based on our review. The first three approaches, Galbraith, forest, and UpSet plots, apply to both binary and categorical subgroup‐defining covariates. We also include two methods, subpopulation treatment effect pattern plot (STEPP) and contour plots, that allow exploring changes in the treatment effect over one or two continuous variables, respectively, as it is suggested in the current EMA guideline.^1^ These five approaches represent or provide a measure of the treatment effect and therefore allow direct comparison across subgroups. Additional graphics that we found less practical are deferred to Appendix available online as Supporting Information while other approaches that may be used to describe subgroup composition but do not fulfil the criterion C1 (effect size) are presented in Supporting Information.

In most of the graphics, for simplicity, the treatment effect is estimated by merely partitioning the dataset and using only subjects from the considered subgroups. We acknowledge that there are more advanced approaches that make more efficient use of the data,^1^ but these are not required to fulfil the purposes of this article (see also Reference 4, 22, and 23). The graphics evaluated in this article can be used to display the treatment effect estimates resulting from such techniques.

All graphics are created using the R statistical software^24^ and the code is publicly available as an R package for reproducibility.^25^ In most of the cases, we draw the plots using functions from the grid and graphics packages which are part of the base R language. For some of the plots, we use additional packages that are cited in each section accordingly.

Although we acknowledge that the choice of colours is an essential and challenging task when producing graphics, we do not discuss this topic in our work as it is discussed elsewhere.^26^, ^27^ Several of the considered plots make use of colour coding to represent the magnitude of the treatment effect across subgroups, for which we use a divergent colour palette generated by the colorspace R package.^28^

We follow Tufte's principles^29^ to enhance graphical integrity. This is particularly relevant when we depict sample sizes with two‐dimensional areas/shapes which are proportional to one‐dimensional sample sizes. Numerical quantities are then properly represented and comparisons can be made accurately. Additionally, we take into account research on graphical perception^30^ to judge the graphics.

#### 2.3.1 Forest plot

**Figure 1:**
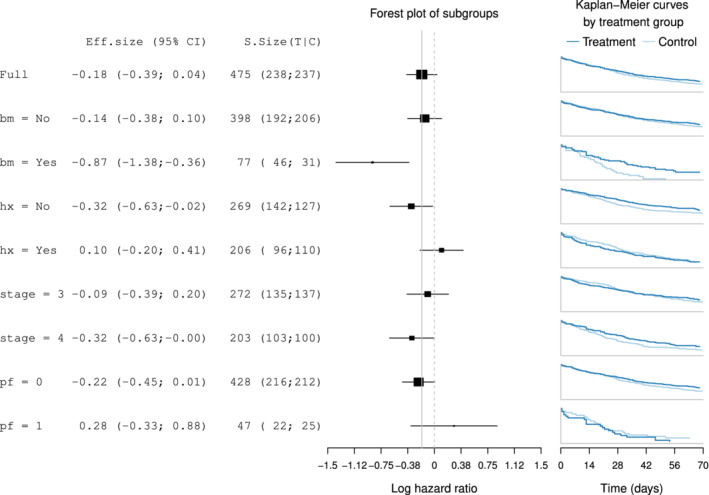
Forest plot for subgroups defined by *performance* (*pf*), *stage*, *history of cardiovascular events* (*hx*), and existence of bone metastasis (*bm*). Effect sizes in terms of the log‐hazard ratio and associated treatment and control group Kaplan‐Meier curves are displayed

Although forest plots are a common graphic used in meta‐analysis,^31^ they are also extensively used for subgroup analysis.^32^, ^33^ Figure 1 shows its application for the prostate cancer dataset considering the four binary covariates. The middle panel displays the subgroup treatment effect estimates with their confidence intervals. The squares in the centre of each error bar are proportional to the subgroup sample sizes. A vertical line at the overall treatment effect level is added to facilitate seeing if a subgroup confidence interval differs significantly from the overall effect.^32^ Additional information in a table format are usually included to provide the magnitude of the estimates. The text in the left panel shows the estimates of the treatment effects, lower/upper bounds of the 95% confidence intervals and subgroup sample sizes (further divided into treatment and control arms). When using continuous endpoints, it is appropriate to display the mean response for each treatment arm in an additional panel. In our implementation for a survival endpoint, we include the Kaplan‐Meier estimate for each subgroup. The summary statistics in the left panel and the survival curves on the right may be dropped if additional space is required. The Kaplan‐Meier estimates are drawn with the ggplot2 package.^34^

Forest plots are popular because they are simple and effective. In the main panel, they allow a direct comparison of the treatment effect estimates with low cognitive effort. According to our assessment, forest plots meet C1 and C2 displaying treatment effects and confidence intervals. Criteria C3 and C5 are also met as the subgroup sample sizes are depicted through the area of the treatment effect and many subgroup‐defining covariates can be easily displayed. A downside of forest plots is that as subgroup intersections (C4) are not shown.

In Figure 1, it is quite clear that the subgroup defined by a positive outcome for *bone metastasis* is the subgroup with the largest benefit from the treatment since the log‐hazard ratio is negative. Interestingly, its upper confidence interval does not cover the average treatment effect, therefore suggesting treatment effect heterogeneity. The Kaplan‐Meier curves also allow to rapidly recognise the differential survival pattern for the subgroup with bone metastasis: patients with bone metastasis in the control group have shorter survival when given the control treatment, while those in the treatment group have a survival pattern that is similar to patients without bone metastasis. For the rest of the subgroups, their treatment effect estimates are closer to the estimate in the overall population. While we observe a positive log‐hazard ratio for some subgroups suggesting the experimental treatment is worse than control, all their confidence intervals cover the average treatment effect, which implies that no treatment effect heterogeneity is present.

#### 2.3.2 UpSet plot

**Figure 2:**
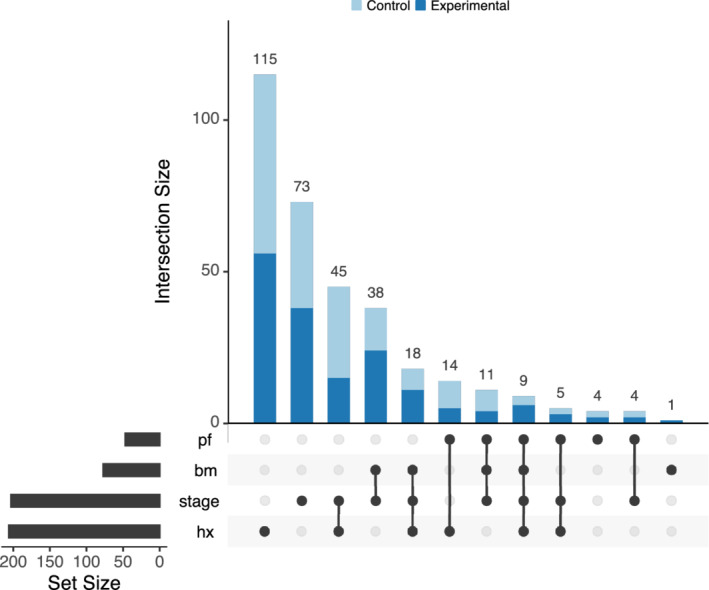
Upset plot displaying the subgroups formed by the intersection of all binary subgroup‐defining covariates

UpSet plots are a novel visualisation technique for the quantitative analysis of sets and their intersections.^35^ It was proposed to overcome the restriction to a small number of sets of Venn diagrams. In Figure 2, we use the UpSetR R package^36^ to create the plot with four binary subgroup‐defining covariates. The sizes of the univariate subgroups for these covariates are shown in the horizontal bar plot in the bottom‐left corner of the figure. The matrix layout on the bottom allows visualising the composition of the subgroup by showing which sets are intersected. The main bar plot displays the sizes of the subgroups that are defined by the respective intersections. For example, the first and tallest bar indicates there are 115 subjects with normal performance rating (*pf* = 0), no existence of bone metastasis (*bm* = 0), *disease stage* 3, and history of cardiovascular events (*hx* = 1). Moreover, we display the number of subjects in each treatment arm for each subset.

**Figure 3:**
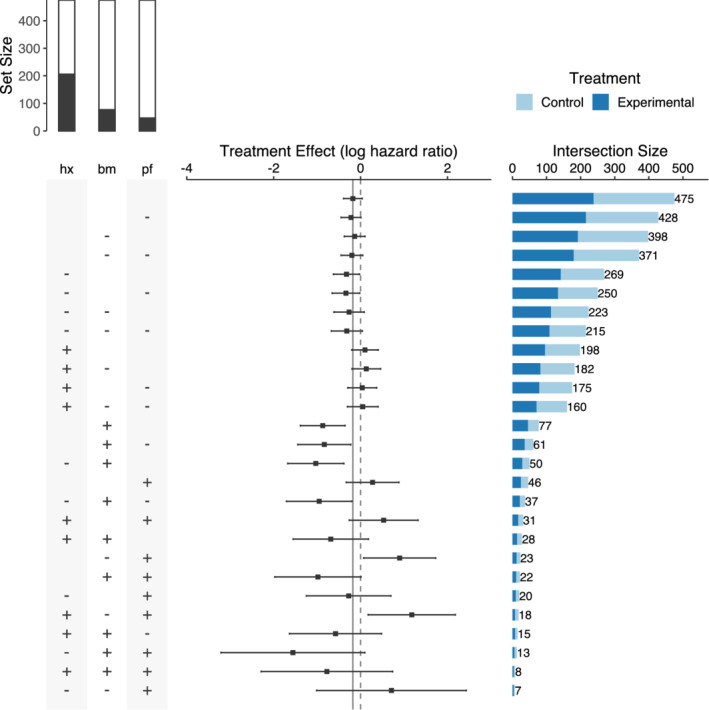
Improved UpSet plot for subgroups defined by *performance* (*pf*), *bone metastasis* (*bm*), and *history of cardiovascular events* (*hx*). The panel on the left (matrix) displays how the subgroups are formed by assigning a “+” if the variable is equal to 1 and a “−” if the variable is equal to 0. The bar plot on top of the matrix panel indicates the marginal set sizes in relation to the total sample size, with the black region corresponding to the 1 or “yes” category and the white region corresponding to the 0 or “no” category. Treatment effect sizes and their confidence intervals are displayed in the panel in the middle and the subgroup sizes in the horizontal bar plot on the right

We extend the UpSetR R package to display effect sizes in an extra panel (Figure 3). While the log‐hazard ratio and its confidence interval for each subgroup are shown as in a forest plot, the UpSet plot provides the advantage of displaying intersections of sets. If one were to use a statistical model with treatment‐by‐covariate interactions to derive the treatment effect estimates, then each row would correspond to a linear combination of the coefficients in the model.

Our extension of the UpSet plot also allows displaying lower level intersections as compared to the original UpSet proposal. We implement a new icon for the matrix panel: a “+” symbol if a variable is equal to 1 or “yes,” a “−” if a variable is equal to 0 or “no,” and empty if this variable is not considered for the subgroup definition. For example, the first bar of the plot corresponds to the overall population (no subgroup division), which has a size of 475. The second bar with a size of 428 corresponds to the subgroup of normal performance rating (*pf* = 0), irrespective of the values of the other two variables. Since the number of subgroups to consider increases dramatically in this modification (3^*p*^ subgroups when considering *p* binary covariates), only three covariates are used. One could include more covariates and filter the number of subgroups according to different criteria, such as total subgroup sample size or sample size per treatment. Finally, the bar plot on top of the matrix panel indicates the marginal subgroup sizes with the black region corresponding to the 1 or “yes” category and the white region corresponding to the 0 or “no” category.

The UpSet plot loses the simplicity observed in forest plots and requires the beholder to be familiar with the graphical approach before drawing conclusions. Nevertheless, the UpSet plot has some advantages. Effect sizes (C1) and confidence intervals (C2) are displayed as in a forest plot and many covariates (C5) can also be used. Compared to a forest plot, subgroup sample sizes (C3) are displayed in a panel as a bar plot. This is a more effective way to display the information in contrast to the proportional areas in the forest plot. Another advantage is that the UpSet plot shows subgroup intersections (C4) and allows inferring relations among the subgroups. In our example, we order the subgroups in terms of their sizes, but it is also possible to arrange the subgroups according to their effect sizes or the number of subgroup‐defining covariates involved in their composition. As the overall treatment effect and its confidence interval are also included, it allows to compare treatment effects and check for treatment effect heterogeneity. However, unlike a forest plot, it does not show the mean response for treatment and control arms in each subgroup.

#### 2.3.3 Galbraith plot

**Figure 4:**
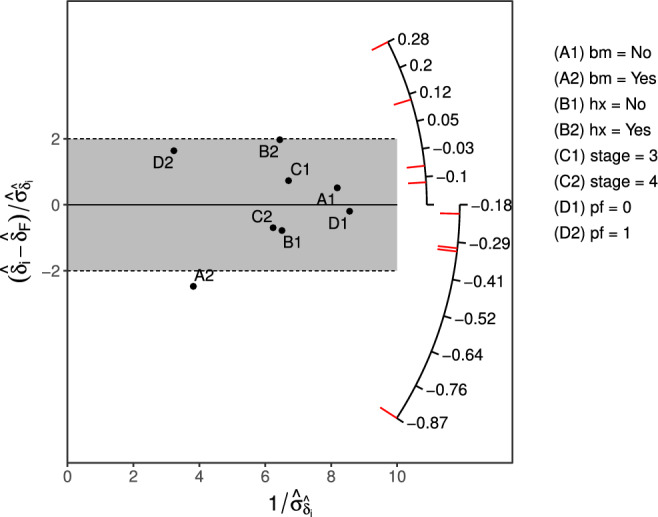
Galbraith plot for subgroups defined by existence of *bone metastasis* (*bm*), *history of cardiovascular events* (*hx*), *stage*, and *performance* rating (*pf*)

A Galbraith plot^37^, ^38^ is an alternative to a forest plot for examining heterogeneity among studies or subgroups in a meta‐analysis. The variant that is shown in Figure 4 exhibits the estimation of treatment effect sizes for *K* = 8 subgroups defined by the four binary covariates. The *xy*‐coordinates correspond to the points:

(1) $$x_{i}=1/\sqrt{\hat{\mathrm{Var}}\left({\hat{\delta }}_{i}\right)},\;y_{i}=\left({\hat{\delta }}_{i}-{\hat{\delta }}_{F}\right)/\sqrt{\hat{\mathrm{Var}}\left({\hat{\delta }}_{i}\right)},$$

where ${\hat{\delta }}_{F}$ is the treatment effect estimate in the full population and ${\hat{\delta }}_{i}$ is the treatment effect estimate in subgroup *i*, *i* = 1, …, *K* . The grey band can be used to detect effect heterogeneity. Points outside the band show larger than expected heterogeneity. The slope of the line from the origin through each subgroup point corresponds to the effect size estimate ${\hat{\delta }}_{i}$ of the corresponding subgroup. An additional radial axis is drawn to depict the subgroup effect sizes which are represented with the red tick marks. The central line at *y* = 0 points to the average treatment effect for the full population. This plot was drawn using the ggplot2 R package together with ggrepel to avoid overlapping labels.

We note that, as ${\hat{\delta }}_{F}$ is itself a random variable, it might better to consider its variance. This can be achieved by considering the *xy*‐coordinates:

$$x_{i}=1/\sqrt{\mathrm{Var}\left({\hat{\delta }}_{i}-{\hat{\delta }}_{F}\right)},\;y_{i}=\left({\hat{\delta }}_{i}-{\hat{\delta }}_{F}\right)/\sqrt{\mathrm{Var}\left({\hat{\delta }}_{i}-{\hat{\delta }}_{F}\right)}.$$

The resulting plot is given in Supporting Information. The drawback of this modification is that the *x*‐axis does no longer represent the standard error of the treatment effect estimates.

The result of the graphical assessment of Galbraith plots is satisfactory, since it displays effect sizes (C1), standard deviations (C2), and a large number of subgroup‐defining covariates can undoubtedly be used (C5). On the other hand, this plot does not display sample sizes (C3) nor intersections (C4). Although Galbraith plots might require more effort to be explained and understood, these plots can certainly handle a large number of subgroup covariates, perhaps better than any of the other considered graphics. In this case, special care needs to be paid to the labels of subgroups and the location of red tick marks as they may not be distinguishable.

In terms of our example, we conclude, just as in the forest plot, that treatment effect heterogeneity may be present in the subgroup of patients with bone metastasis since its point is immediately visible outside the grey band.

#### 2.3.4 Subpopulation treatment effect pattern plot

**Figure 5:**
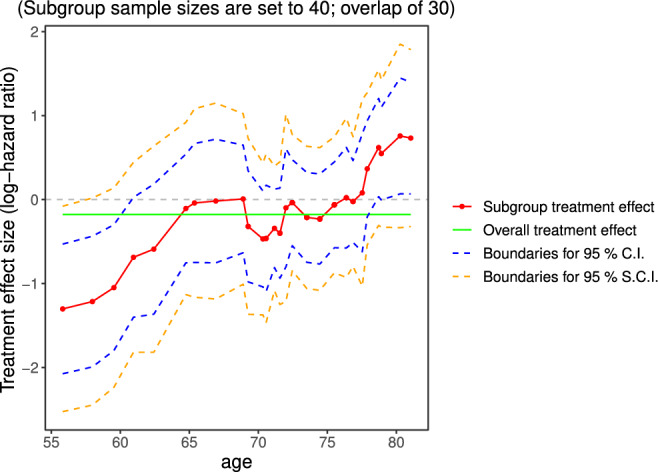
STEPP plot of overlapping subgroups defined by age. Each subgroup has a sample size of around *N*_11_ = 40 and is controlled to have about *N*_12_ = 30 subjects overlapping with the neighboring subgroups. STEPP, subpopulation treatment effect pattern plot

The STEPP^39^, ^40^ gained popularity in breast cancer recently. It is a non‐parametric method mainly for examining whether treatment‐covariate interactions exist. In Figure 5, we adopted the slide‐window fashion of STEPP to represent the estimation of treatment effect size (log‐hazard ratio) in overlapping subgroups defined by *age*. To do so, we form subgroups with sample sizes of around *N*_11_ = 40 with an overlap of *N*_12_ = 30 subjects with immediately neighbouring subgroups. The band bounded by the blue dashed lines is constructed for 95% simultaneous confidence interval. The other band bounded by the orange dashed lines is built based on individual 95% CI (without multiplicity adjustment). The red line is formed by connecting the point estimates of treatment effect (log‐hazard ratio) for all formed subgroups. The green line represents the log‐hazard ratio estimate for the full patient population. It is worth noting that the point estimates are positioned at the mean value of *age* for each subgroup for the *x*‐axis. If the green line does not lie in the region formed by simultaneous confidence intervals, it reveals that interaction may exist.

In the original publication,^40^ the points were placed equidistantly along the *x*‐axis annotating the median values of the variable for each subgroup as reference. An illustration of this alternative plot is given in Supporting Information. We believe it is better to use the proper scale to reflect the mean (or median) values of the variable used to define subgroups. This helps indicate whether the values cover a small or large range of the variable of interest.

It is a quite common problem in subgroup analysis to define subgroups based on continuous biomarkers. Since it is advised against using arbitrary cutoff points in initial subgroup investigations, STEPP plots are a good way to characterise changes of the estimated treatment effect over the range of the considered continuous covariate. This is the suggestion from the current EMA Guideline on the investigation of subgroups in confirmatory clinical trials.^1^

The STEPP approach satisfies C1 displaying effect sizes and C2 for displaying confidence intervals. Here, the subgroup sample sizes (C3) are adopted by design and only annotated in the figure title but are not represented graphically. This plot only considers one continuous covariate and therefore, C4 (intersections) cannot be met. The plot does show intersections of contiguous subgroups, where the total number of subgroups depends on the sample size of subgroups and the overlap proportions.

In some situations, it might not be clear how to determine the subgroup sizes or overlap and sensitivity analyses might need to be conducted for different configurations. The analysis results may further be compared with the results when using fractional polynomials^41^, ^42^ or non‐parametric methods such as Gaussian processes.^43^

In Figure 5, we observe that the treatment effect for subgroups defined by *age* fluctuates closely around the overall treatment effect. When approaching the ends of the range of the covariate the estimate of the log‐hazard ratio departs from the estimate for the full population although the confidence intervals for the subgroup treatment effects still cover the overall effect. This graph may be particularly useful to derive subgroups from a continuous variable.

#### 2.3.5 Contour plot

While STEPP considers only one continuous biomarker, a contour plot could be regarded as an extension to explore continuous changes in two continuous biomarkers. We propose two different implementations of contour plots for the treatment effects across *age* and *weight*.

**Figure 6:**
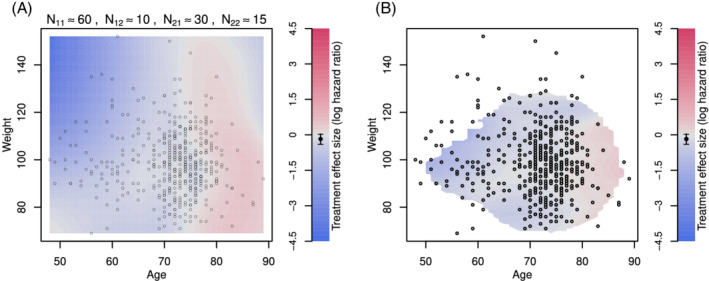
Contour plot of treatment effect in terms of the log‐hazard ratio over the plane of *age* and *weight*. A, Contour lines are drawn by forming subgroups with neighboring subjects, calculating the treatment effect for subgroups and interpolating the results using loess. *N*_11_ stands for the sample size of a marginal subgroup defined by a range of *age*, *N*_12_ is the overlap size of the immediate marginal subgroups on *age*, *N*_21_ is the sample size of the subset of a marginal subgroup on *age* but further defined by a range of *weight*, and *N*_22_ is the overlap size of the immediate subgroups (which are the subset of a marginal subgroup on *age*) on *weight*. B, Contour lines are drawn by fitting a local regression at each point of the grid, using subjects weights according to their distance to the point of the grid. Points with few subjects in the vicinity of the grid point were left blank

In Figure 6A, subgroups of sample sizes *N*_11_ are formed by using a horizontal sliding window across the values of *age* with an overlap of *N*_12_ subjects. Subsequently, each subgroup is further divided into smaller subgroups of sample sizes, *N*_21_, using a vertical sliding window across the values of *weight* with an overlap of *N*_22_. Sample sizes and overlap to form subgroups are adopted by design based on sensible judgement. For example, subgroups should have a considerable sample size to ensure that patients in both treatment and control arms are represented. For each formed subgroup, we then calculate the log‐hazard ratio for treatment vs control. The contour areas are obtained through a bivariate interpolation and smooth surface fitting (LOESS) for irregularly distributed data points over the range of values from the subjects under study. A divergent colour scale is used for the effect sizes. A limitation of this approach is that there may be regions of the covariate space in which the treatment effect estimates are not reliable due to small sample sizes or no data points.

We also propose using local regression techniques to calculate the treatment effect at each coordinate. In Figure 6B, a weighted Cox proportional‐hazards model is fitted at each combination of *weight* and *age* (using a step of 1 unit). A normal kernel with the centre at the coordinate values under consideration is used to assign weights to each subject. If there are less than 20 subjects within two standard deviations, the effect size is not calculated and the area is left blank. This helps to avoid extrapolating the results to areas in which we do not have enough information.

Contour plots match criterion C1 since effect sizes are represented through a colour scale which is one of the least accurate ways to encode information.^30^ This is because for a particular coordinate in the plot, it might be hard to decipher what is the precise value of the treatment effect. This plot helps to uncover patterns in specific regions of continuous covariates that might not be visible otherwise. Contour plots also meet C4 as the intersection of two subgroup‐defining covariates are used. The uncertainty of the treatment effect estimates (C2) and sample sizes (C3) are not represented in the graphic which is a significant drawback. Contour plots only consider two covariates.

Contour plots are particularly useful when there are enough subjects well distributed over the entire range of the covariates of interest. The interpolated treatment effect sizes may be unreliable in regions where there are no data points. In situations where the values of two covariates are sparsely distributed over the region, it can be unclear how smooth the interpolated surface should be. Note that it is also possible to use other local regression algorithms to calculate the treatment effect at each coordinate or even other modelling strategies such as including a generalised additive model with interactions.^41^ Recent proposals that investigate the predicted individual treatment effect can also be applied to estimate the effect of treatment across the covariate space.^44^, ^45^, ^46^

We observe that older patients seem not to benefit from the new treatment. However, this interpretation should be cautious as the precision of the estimates is not displayed.

### 2.4 Additional graphical approaches

We also consider further graphical approaches that may be applied to the subgroup analysis framework: level plot, mosaic plot, Venn diagram, bar chart, tree plot, L'Abbé plot, chord diagram, and Coxcomb plot. Compared with the aforementioned methods, their assessment is less favourable and hence they are only presented in Appendix available online as Supporting Information. In most of the cases, they convey the information of treatment effect through colour coding. This way of presenting the information is more challenging to decode. Additionally, most of them do not display a measure of uncertainty for the treatment effect estimates which is essential for assessing treatment effect heterogeneity.

The use of auxiliary plots might help to display additional information, such as overlap between subgroups, that might be relevant. The Supporting Information provides an overview of some options. Some of the graphics allow visualising subgroup composition or overlap between subgroups by displaying the relative overlap or dissimilarity measures. Other graphics, such as a mosaic plot with a binary response, an alluvial plot or a coxcomb plot, may complement the analysis by displaying absolute response rates in treatment and control arms across subgroups.

## 3 SUBGROUP ANALYSIS SUMMARY

### 3.1 Summary of case study: The prostate cancer dataset

Throughout the manuscript, we have analysed the prostate cancer dataset to explore subgroup effects. Here, we present an overall summary of the main findings related to subgroups.

In the forest plot (Figure 1), we explored the marginal treatment effects for subgroups defined by binary covariates. The treatment effect was similar across all the subgroups except for the group of patients with *bone metastasis*. The graph suggests that patients with *bone metastasis* might have larger benefit from the experimental treatment because the confidence interval for this subgroup does not cover the line that represents the treatment effect in the overall population. The same pattern is observed using a Galbraith plot (Figure 4), as the only point lying outside the (−2, 2) band is the one corresponding to this subgroup.

Figure 3 allows, in addition, to observe subgroups formed by the subgroup intersections. It can be seen that patients without *bone metastasis* and with a *history of cardiovascular events* might have been harmed by the experimental treatment.

The variable *age* was explored alone in Figure 5 and together with *weight* in Figures 6. In the latter, we find that the treatment appears more beneficial for younger patients with *weight* index above 90, while for older patients the treatment may have led to worse outcomes than control.

We remind here that these analyses are exploratory and must be interpreted with care. Despite this, they may bring useful insights to plan additional studies and collect more information from subgroups of interest in the future.

### 3.2 Summary of graphical methods

In this section, we provide a summary regarding the criteria C1 to C5 presented in Table 1. We discuss only the graphics presented in the previous section. The assessment and characteristics of the improved graphical approaches are summarised in Table 2 where we also include the graphics in Appendix available online as Supporting Information.

**Table 2:** The assessment summary of graphical techniques for subgroup problems

	Criterion					Additional features	
	C1	C2	C3	C4	C5	T/C effect	Covariate type
Galbraith plot^a^	*✓*	*✓*			*✓*		B/Cat
Forest plot^a^	*✓*	*✓*	*✓*		*✓*	*✓*	B/Cat
UpSet plot^a^	*✓*	*✓*	*✓*	*✓*	*✓*		B/Cat
STEPP	*✓*	*✓*					Cont
Contour plot	*✓*			*✓*			Cont
Tree plot	*✓*	*✓*		*✓*			B/Cat
Level plot	*✓*		*✓*	*✓*			B/Cat
Mosaic plot	*✓*		*✓*	*✓*			B/Cat
Venn diagram^a^	*✓*		*✓*	*✓*			B
Bar chart	*✓*		*✓*	*✓*			B/Cat
L'Abbé plot^a^	*✓*		*✓*		*✓*	*✓*	B/Cat
Chord diagram	*✓*		*✓*		*✓*		B/Cat
Coxcomb plot	*✓*		*✓*	*✓*			B/Cat

*Note:* The assessment criteria are: C1: effect size; C2: uncertainty; C3: sample size; C4: intersections; C5: many covariates. T/C effects stands for displaying the average response in treatment and control arms. Covariate types are B = binary, Cat = categorical, Cont = continuous. Graphics in Appendix available online as Supporting Information are also included in this table for comparison purposes.

Abbreviation: STEPP, subpopulation treatment effect pattern plot.

a The plot has been improved or modified to make it available for the subgroup analysis framework.

*C1* (*effect size*): This information is encoded in different ways in the studied graphics. Forest plots, UpSet plots, Galbraith plots, and STEPP allow a straightforward comparison across subgroups as the treatment effect estimates are illustrated along a common axis. This way of encoding information is the most accurate according to theoretical arguments and experimental results on graphical perception.^30^ Contour plots use a less accurate encoding that is effective to only give a general overview of the estimated treatment effect over the range of the covariates. Therefore, even if all of the graphical techniques satisfy the primary criterion of displaying subgroup treatment effect sizes, some may be more effective in communicating the results from the analysis than others. The judgement of heterogeneity also depends on the treatment effect estimate in the full population, which is displayed in all the considered graphics. Additionally, forest plots can provide absolute subgroup responses for the treatment and control arms.

*C2* (*uncertainty*): Forest plot, STEPP, and UpSet plot display the confidence intervals of the treatment effects while Galbraith plot shows their standard error. This is important since visualisations that do not adequately demonstrate the uncertainty in the estimates may be misleading and can lead to an over‐interpretation of the heterogeneity among subgroup effects.

*C3* (*sample size*): Only UpSet plot and forest plot provide a visual display on subgroup sample sizes. The UpSet plot displays the subgroup sample sizes in an additional panel using a bar plot which allows more efficient and accurate comparison of subgroup sizes in contrast to the forest plot. While one can add a bar plot showing sample sizes to any of the other graphics, the particular assembly of the UpSet plot enables to decode the information quickly and efficiently.

*C4* (*intersections*): This criterion is only met for UpSet plots and contour plots. UpSet plots can display intersections of two or more subgroups remarkably, allowing great flexibility in how the information is presented.

*C5* (*many covariates*): Forest plots, Galbraith plots, and UpSet plots can display a large number of subgroups‐defining covariates. However, Galbraith plots should be highlighted in this criterion as its design makes it more appropriate when considering a large number of covariates.

## 4 DISCUSSIONS AND CONCLUSION

We made use of several graphical approaches and assessed their characteristics for subgroup problems. We also attempted to improve some methods correcting flaws or adapting graphics for the subgroup analysis setting.

It is important to note that the considered graphical approaches are descriptive only and do not adjust for potential selection bias of point estimates, inflated type 1 errors due to multiple testing, or reduced simultaneous coverage probabilities of confidence intervals. These consequences of multiple testing and selective estimation may become substantial as the number of considered subgroups increases. In exploratory settings where the definition and selection of subgroups are post‐hoc and may be data‐driven, frequentist error rates or coverage probabilities cannot be controlled anyway. In contrast, if the subgroups to be considered are pre‐defined (or selected independently of outcome data) there is a broad range of statistical approaches available to account for the associated multiplicity.^3^, ^47^ Most of the considered graphical approaches can be used to show multiplicity adjusted treatment effects and uncertainty measures. One can, for example, use simultaneous confidence intervals based on the Bonferroni correction, post‐selection confidence intervals,^46^ treatment effects estimates after model averaging,^48^ bias‐adjusted estimates,^21^ and so on. Comparative plots showing both the adjusted and unadjusted estimates may also provide valuable insights.

In this article, we provide tools to visualise essential information on subgroups, as effect size estimates and subgroup sample sizes. The considered approaches are descriptive only and serve as exploratory tools for hypotheses generation for future investigations.

The choice of the visualisation method depends on: the type of biomarkers that define the subgroups, the type of outcome variable, the sample sizes, and the objective of the subgroup analysis. For example, we have seen that contour plots and STEPP are only suitable for continuous covariates, while the other plots allow the use of binary or categorical covariates. On the other hand, Galbraith plots might be particularly suited for the case of a very large number subgroups and Forest plots may show not only the treatment effect estimates but also the average response in each treatment arm. As some graphics do not display all information, combining several plots can be advantageous.

In this work, we focused on non‐interactive graphical displays. We recognise the usefulness of adding interactivity which can improve the flexibility of the studied graphics. For example, there exist work on interactive mosaic plots^49^ which allows easy inclusion of many subgroup‐defining covariates avoiding the problem of overlapping labelling. Interactive UpSet plots allow inclusion/exclusion of covariates, ordering them according to different characteristics, and displaying additional variables; which makes this graphic a powerful analysis tool (https://caleydo.org/tools/upset/). Galbraith plots might benefit from interactivity when using a large number of covariates, by using mouse hover over the points to display the corresponding labels and subgroup effect sizes. The recently published subscreen package^50^, ^51^ enables the analysis of thousands of subgroups by using a scatter plot and allowing the user to display additional information thanks to interactive tools like the Shiny R package.^52^ Existing interactive approaches can be adapted to subgroup analysis, or interactivity can be added to the graphics introduced in this article.

Finally, the dataset we used for illustration contained information on causes of death. However, the considered endpoint in the analysis in this article was death from all causes combined. Additionally, while four treatment options were used to treat the patients, we combined them into two categories. These adaptations allowed us to frame the analysis in the typical situation where an experimental treatment is compared against a control. Modifications to the considered graphics could be explored to enable the comparison of multiple treatments or multiple endpoints. Again, interactivity may help in these situations to explore and understand the data.

## Supporting information


**Appendix** S1: Supporting Information

## Figures and Tables

**Figure A1:**
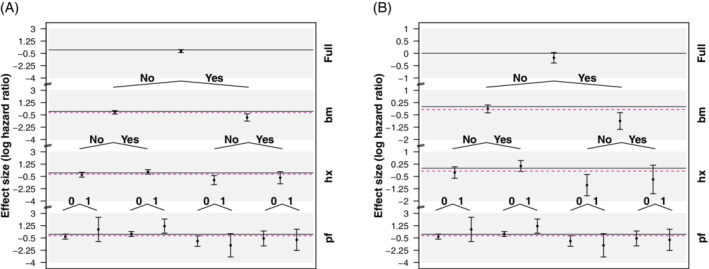
Tree plot for the treatment effect in terms of the log‐hazard ratio for subgroups defined by category combinations of existence of *bone metastasis* (*bm*), *history of cardiovascular events* (*hx*), and *performance* rating (*pf*). Each layer shows the 95% CI of treatment effect differences for the associated subgroups. The purple horizontal lines placed in the middle of CI have a length proportional to the relative subgroup sample size over the full population. In A, the *y*‐axes are independent in each layer of the plot, while in B, *y*‐axes are kept fixed across levels, which allows comparing variability in the estimates

**Figure A2:**
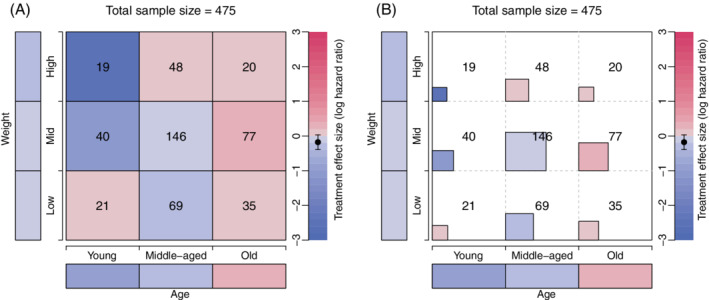
Level plots of treatment effect in terms of the log‐hazard ratio across mutually disjoint subgroups defined by *age* and *weight* categorised in three levels. The cells on the bottom and the left margins correspond to the marginal subgroups defined by the levels of *age* and *weight*. In B, the area of each square inside the cells is proportional to the sample sizes, which are also displayed in the middle of the cells

**Figure A3:**
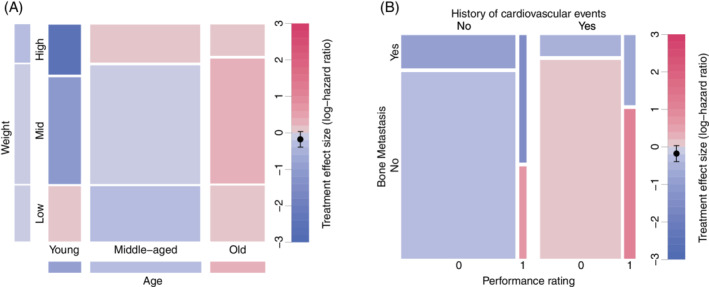
A, Mosaic plot of treatment effect in terms of the log‐hazard ratio across mutually disjoint subgroups defined by *age* and *weight* categorised in three levels. The cells on the bottom and the left margins correspond to the marginal subgroups defined by the levels of *age* and *weight*. The area of each mosaic is proportional to the sample sizes. B, Mosaic plot of treatment effect in terms of the log‐hazard ratio across mutually disjoint subgroups defined by *history of cardiovascular events*, *performance rating*, and *bone metastasis*

**Figure A4:**
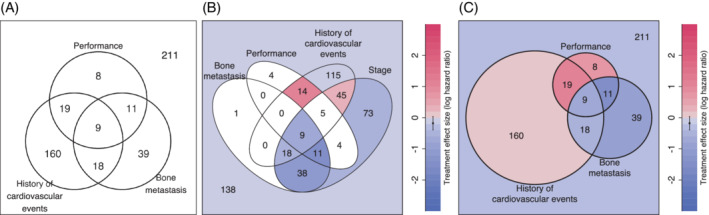
A, Venn diagram of three subgroups defined by presence of *bone metastasis*, *history of cardiovascular events*, and *performance* rating = 1. B, Venn diagram of four sets defined by presence of *bone metastasis*, disease *stage*, *performance* rating = 1, and *history of cardiovascular events* with treatment effect sizes in terms of the log‐hazard ratios. C, Approximate area‐proportional Venn diagram of three subgroups defined by presence *bone metastasis*, *history of cardiovascular events*, and *performance* rating = 1 with treatment effect sizes in terms of the log‐hazard ratios

**Figure A5:**
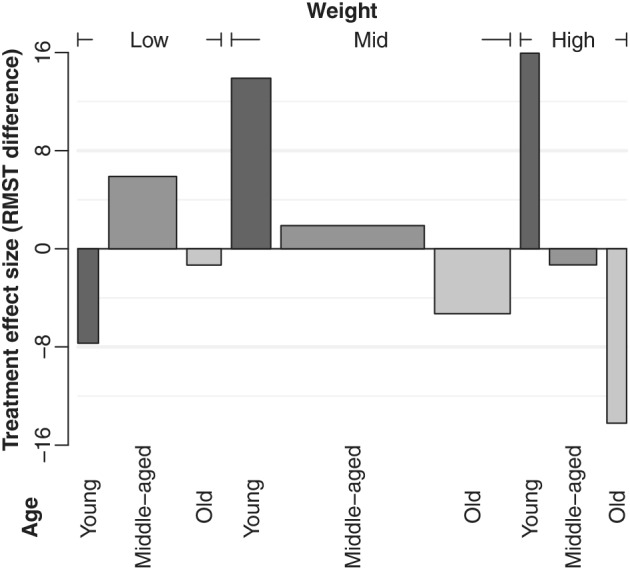
Bar plot of treatment effect in terms of the difference in restricted mean survival time across mutually disjoint subgroups defined by *age* and *weight* categorised in three levels. The width of each bar is proportional to the sample size for subgroups. The area can be interpreted as the gain/loss in restricted mean survival when using treatment in comparison to control. Black, grey, and light grey indicate the *age* categories young, middle‐aged, and old, respectively

**Figure A6:**
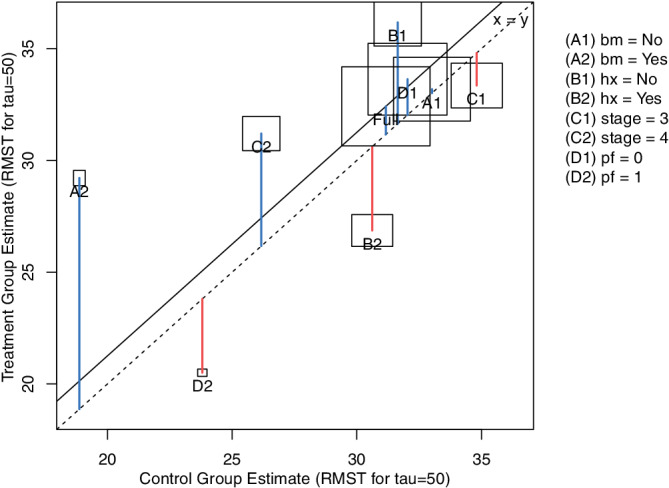
L'Abbé plot for subgroups defined by *performance* (*pf*), *stage*, *history of cardiovascular events* (*hx*), and existence of bone metastasis (*bm*). Effect sizes are given in terms of the difference in restricted mean survival time (RMST)

**Figure A7:**
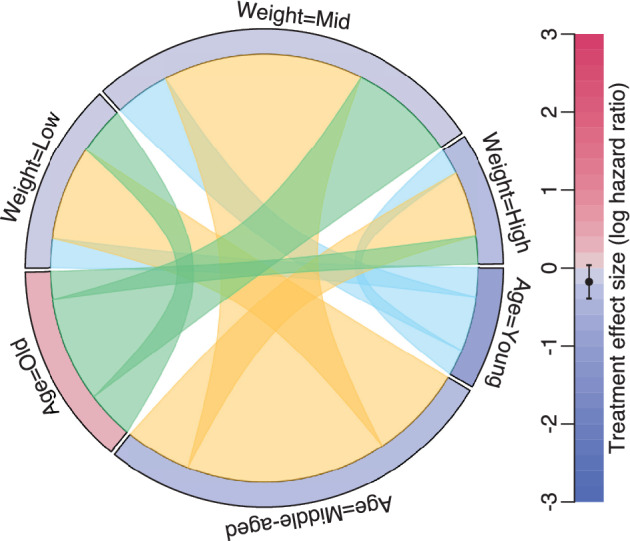
Chord diagram for the subgroups formed by *age* and *weight*. The colours along the circle represent the treatment effect in terms of the log‐hazard ratio. The ribbons that link the subgroups represent their overlap

**Figure A8:**
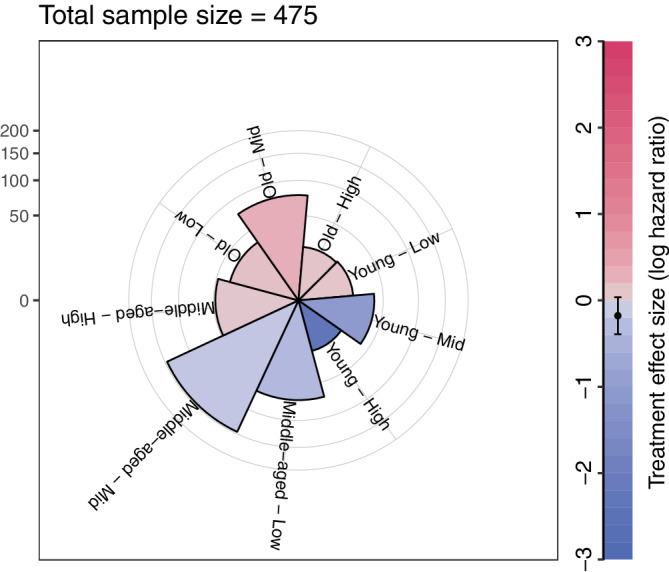
Nightingale coxcombs plot for subgroups defined by *age* and *weight*. The colours represent the treatment effect in terms of the log‐hazard ratio. The radius of the sectors are proportional to the square root of the sample sizes in the subgroups

## Data Availability

The code and data used to generate the figures in this article are provided in the Supporting Information together with an R package, which is also available on CRAN (https://cran.r-project.org/package=SubgrPlots).

## Appendix A: Further visualisation methods 1

### 1.1

We present here alternative plots that might be used for subgroups analysis but we found less practical. Except for tree plots, they do not show the uncertainty of the estimates and may be misleading for that reason.

For some of the plots in this Appendix, we categorise the *age* and *weight* covariates into three levels to create subgroups (*age*: young = [48, 65], middle‐aged = (65, 75], old = (75, 89]; *weight*: low = [69, 90], mid = (90110], high = (110152]). We note that this is for illustrative purposes only to show examples with ordinal variables. Any real analysis of continuous covariates should not categorise them but treat them as continuous.^41^, ^53^

#### A1 Tree plot

The tree plot for subgroup analysis starts with the full population that branches into two or more items corresponding to the levels of the first subgroup‐defining covariate. Each of the items in the new level branch again into two or more levels for the second covariate and so on. If more variables were included, this division procedure is consecutively conducted to form subgroups until all the category combinations of the covariates are considered. Figure A1 shows a tree plot of treatment effect differences for subgroups defined by *bone metastasis*, *performance rating*, and *history of cardiovascular events*. In each level or layer, treatment effect differences and their 95% confidence intervals for the associated subgroups are displayed. An additional horizontal dotted line is added at each level for the overall treatment effect size. In Figure A1a, the *y*‐axis for each level of the plot is drawn independently from the other levels. In Figure A1b, the *y*‐axes are consistent across levels, which help to visualise the difference in variability of the estimates.

Tree plots display effects sizes and their confidence intervals satisfying C1 and C2. This information is encoded through the position on identical but nonaligned scales, which provide a less accurate perception when compared to the forest and UpSet plots. Tree plots allow displaying the intersection of not only two but also more subgroup‐defining covariates (C4). However, they do not show the size of the subgroups (C3) and it is not possible to arrange many subgroup‐defining covariates (C5).

It is worth pointing out a few features of tree plots. Although we used binary covariates, it is possible to consider covariates with more than two levels. Ideally, the number of covariates and categories should be moderate or we may have subgroups with small sample sizes. In this implementation, the ordering of the covariates needs to be pre‐specified. Recent proposals that allow the data to define the ordering and/or cutoff values for continuous variables^54^, ^55^ can be used to draw tree plots.

Figure A1 allows us to draw additional conclusions regarding the treatment effect sizes. We observe that the treatment effect is more pronounced for subjects with bone metastasis. Additionally, we notice that the subgroup of subjects without bone metastasis but with a history of a cardiovascular event and limited activity (*pf* is 1) has a positive log‐hazard ratio suggesting that the control is better than the experimental treatment for this subgroup.

#### A2 Level plot

Level plots are typically used to show geographic surfaces in a plane. In the subgroup analysis setting, two categorical variables are arranged on the axes and the main plot area consists of cells that represent disjoint subgroups. Each subgroup is defined by the corresponding combination of levels of both covariates and a divergent colour scale is used to display the treatment effect in that subgroup. In Figure A2a, we show the implementation of a level plot for treatment effects in terms of log‐hazard ratios in subgroups defined by the categorised *age* and *weight* for the prostate cancer dataset. For each subgroup, a Cox proportional hazards model with treatment as the independent variable is fitted to obtain the estimate for the hazard ratio. Alternatively, a single multivariate model with treatment by subgroup interactions may be fitted to obtain effect estimates. We also add the point estimate and confidence interval for the overall population in the legend as a reference and include the subgroups’ sample sizes inside the cells. The cells on the bottom and the left margins represent the marginal subgroups corresponding to each of the three levels of *age* and *weight*, respectively.

This graphical approach satisfies criterion C1 displaying effect sizes. A quick look at the colours allows conclusions such as for which subgroups the treatment is beneficial and for which ones it is harmful. However, this way of encoding quantitative information provides the least accurate visual perception and it is hard to compare between subgroups with similar treatment effect. Additionally, the variability of the subgroup estimates is not represented in this plot (C2) therefore making it impractical to detect treatment effect heterogeneity. Although the addition of the sample sizes in the cells allows a comparison of the subgroup sizes, the sample sizes are not represented by the figure therefore this display meets criterion C3 only partially. Level plots display the intersection of the subgroups formed by the levels of the subgroup defining covariates (C4). It is worth noting that only two covariates can be considered in a level plot. Finally, we remind that because the cutoff points for continuous covariates may be arbitrary, level plots are best suited for categorical covariates.

Examining Figure A2, we may conclude that the treatment is worse for older patients and young patients with low weight as the direction of the treatment effect is reversed. Moreover, the treatment seems to be even more beneficial for heavier young patients. These interpretations need to be taken with care as the precision of the estimates is not given and the small sample sizes in some subgroups may lead to highly variable effect estimates.

As a possible improvement, the coloured squares inside each cell are drawn with areas proportional to the subgroup sample sizes (Figure A2B). This allows comparing subgroup sample sizes more easily. At the same time, it may be difficult to see the colour in each square, particularly in the case of small sample sizes. Perhaps a better way to present the information of the level plot is using a mosaic plot as described in the following section.

#### A3 Mosaic plot

Mosaic plots are useful to represent contingency tables by arranging proportional‐to‐size cells in a grid. There are some variations in which this type of plot may be used in subgroup analysis. First, we devise an improvement of the level plot as in Figure A3A. Although the sample size annotation in each mosaic could be easily added, we omit it here as the sample sizes are depicted through the area of the mosaics. The interpretation of this plot is similar to the level plot presented in Figure A2B.

Mosaic plots offer the advantage that more covariates can be included. In Figure A3B, we use *history of cardiovascular events*, *performance* , and *bone metastasis* to illustrate a mosaic plot with three subgroup‐defining covariates. As a drawback, when adding additional covariates, it is no longer possible to show the information on marginal subgroups.

Figure A3B allows us to observe that there may be heterogeneity in the treatment effect as some subgroups have effect estimates in the positive direction while others in the negative direction. However, the absence of uncertainty measures for the treatment effects estimates prohibits conclusive interpretation.

#### A4 Venn diagram

Venn diagrams are undoubtedly the most widely used tool to visualise sets and their relations. In the subgroup analysis setting, Venn diagrams may be used to display the composition of a dataset. A Venn diagram for subgroups defined by *bone metastasis*, *history of cardiovascular events*, and *performance* is shown in Figure A4A. Each circle defines the subgroup of patients for which the level of the corresponding variable is “yes” or 1. The diagram indicates the sample sizes for all the subsets that are formed by set operations (intersection and complement) on the three subgroup‐defining covariates. The number outside of the three circles indicates the size of the complement of the union of the three subgroups.

Figure A4B,C considers Venn diagrams with four and three subgroup‐defining covariates respectively. Both encode the treatment effect in terms of the log‐hazard ratio by colouring the corresponding regions. This feature thus enables the Venn diagram to satisfy criterion C1. The variability of the estimates is not given and therefore C2 is not met.

As seen in Figure A4B, using four ellipses for representing all possible subgroups (formed through intersection and complement) is visually appropriate. Other shapes (such as polygons^56^, ^57^) can be used but the visualisations may not be easy to understand. In our example, we obtain subgroups with small sample sizes when considering the intersections of the four covariates. The white regions indicate that it is not possible to calculate the treatment effect in the corresponding subgroup. An additional rule may be added to this plot to colour only the areas that attain a pre‐specified sample size.

Figure A4C considers proportional‐area methods where each covariate representative region area is proportional to the respective sample size proportion. The region areas only approximately correspond to the sample size proportions because of the limited degrees of freedom for circles. We employ the simple algorithm mentioned in Reference 58. Other algorithms to display each region area proportional to the sample sizes are available. Recently, an algorithm that can produce accurate area‐proportional Venn diagrams using ellipses was developed.^58^ However, the algorithm is somewhat sophisticated and only works on three sets.

Venn diagrams are implemented using the VennDiagram R package^59^ together with the polyclip package.^60^ For proportional‐area Venn diagrams, we further use the sp package^61^ and the rgeos package.^62^

Venn diagrams satisfy C3 (sample size) and C4 (intersections) in our assessment. However, as in level and mosaic plots, the encoding is not optimal and the UpSet plot provides a better alternative. Useful extensions to Venn diagrams, such as the Edwards’ construction^63^, ^64^ are available so that they can accommodate a larger number of covariates. The total number of subgroups including mutually disjoint groups can be 2^*p*^, where *p* is the number of binary covariates considered. Despite this merit, there is a limit on the number of the sets considered in practice. It may become complicated to interpret a Venn diagram with more than five subgroup‐defining covariates.

Figure A4 shows that the treatment effect is reversed for those subjects without bone metastasis when they have previous cardiovascular events or limitation of activity (*performance rating* is 1).

#### A5 Bar chart

Another graphical technique to depict treatment effect sizes is a bar chart. They are easy to interpret and allow direct comparison among subgroups. For the subgroup analysis problem, we use subgroups defined by the level categorisation of *age* and *weight* variables used in the previous examples and consider the difference in restricted mean survival time (RMST) as treatment effect instead of the hazard ratio. In Figure A5, each covariate is categorised into three levels and the bars represent mutually disjoint subgroups. The levels of *age* and *weight* are respectively listed at the top and bottom part of the figure. The height of the bars is proportional to the treatment effect differences between the treatment and control arms, that is, the difference in RMST. The width of the bars is proportional to the subgroup sample sizes. This arrangement has another useful property; the area of the bars is proportional to the restricted mean survival gain or loss in each subgroup when using the experimental treatment in comparison to control. Different variations of grey were used to show which subgroups have the same category level on *age*.

Based on our assessment, this graphical representation approach holds C3 (sample size), C4 (intersection) but not C2 (uncertainty), and C5 (many covariates). Each bar is the intersection of two subgroups defined by *age* and *weight* with their respective levels. Such a graphical approach does not allow examining heterogeneity in treatment effects across subgroups as the overall effect size and the variability in the subgroup effect estimates are not shown.

Few noteworthy characteristics also need to be mentioned. If considering more covariates, one could label all the level combinations of the covariates in the bottom part of the picture or simply to make a legend elsewhere. However, a high number of covariates or levels may be problematic, making it difficult to compare the widths of the bars. Second, as in level plots, the cut‐off points for categories in continuous variables may be arbitrary and categorical covariates are therefore preferred for bar plots.

Although we use a different measure for the treatment effect, the direction of the estimates is maintained compared to the level plot in Figure A2 and the interpretation remains unchanged.

#### A6 L'Abbé plot

L'Abbé plots^65^ are a variant of scatter plots which are useful for examining heterogeneity in a meta‐analysis. The graphic is originally intended for binary outcome data to represent risk ratios, risk differences, or odds ratios between treatment and control. For our implementation, we extend this graphical technique to the case of continuous and survival outcomes and also modify points to rectangles (Figure A6). The *xy*‐coordinates for each subgroup correspond to the estimates of the RMST in the control and treatment arm, respectively. The width and the height of a rectangle (corresponding to a subgroup) respectively indicate the sample sizes of the control and treatment arms in the subgroup. We draw a diagonal dashed line at *y* = *x* which represents no treatment effect (equal RMST in both arms) and a solid diagonal line with *y*‐intercept at the overall treatment effect size. Each rectangle has a vertical segment from its centre to the diagonal dash line representing the magnitude of the effect size, that is, the gain (in blue) or loss (in red) in terms of RMST when comparing treatment vs control.

L'Abbé plots satisfy C1 (effect size), C3 (sample sizes), and C5 (many covariates), but they do not show the uncertainty of the treatment effect estimates (C2) nor subgroup intersections (C4). While they may handle many subgroups, it may be difficult to untangle the corresponding rectangles if subgroups have a similar effect estimate for treatment and control groups.

This graphical tool allows us to draw an additional conclusion in our example. The subjects with bone metastasis in the control group have a lower RMST compared to other subgroups. When receiving the experimental treatment, the RMST is closer to that in other subgroups.

#### A7 Chord diagram

Chord diagrams are widely used to visualise genomic data.^66^ There are several approaches to these diagrams although the main aspect is that they allow representing the relationships between pairs of sets. For our example, we use the categorised variables *age* and *weight* (Figure A7). The categories of each variable are arranged along the circle where each of their corresponding cells has a size proportional to the corresponding subgroup sample size and a colour representing the treatment effect estimate in terms of the log‐hazard ratio. The ribbons in the centre of the diagram represent the relative overlap between the categories of the variables. Their width is calculated in correspondence to the proportion of subjects from a subgroup that is also in the subgroup to which the bands connect. We implement this graphic using the circlize R package.^67^

The flexibility of this plot is an advantage since many other implementations may be devised, especially when the number of covariates is extremely large as when dealing with genomic data (C5). However, while chord diagrams display the effect sizes (C1) and sample sizes (C3), other alternatives might be more effective for the analysis of treatment effects of subgroups. The treatment effects for the intersection of subgroups is not displayed (C4) but chord diagrams show the overlap between subgroups which helps in clarifying that we look at the subgroups are not disjunctive. Their main disadvantage is that no uncertainty measures of the treatment effect estimates are displayed (C2).

Figure A7 allows us to observe the treatment effects across the subgroups defined by *age* and *weight* marginally. Since the direction of the treatment effect changes across the levels of the *age* covariate, treatment effect heterogeneity may be present. Again, using a colour scale and not displaying variability estimates hinders a definite conclusion.

#### A8 Coxcomb plot (Nightingale rose)

A Nightingale coxcomb plot^68^ is a type of radial plot that was introduced in 1858 and is usually recommended as an alternative to pie charts.^9^ In Figure A8, we arrange the subgroups defined by the categorised *age* and *weight* variables along the circle using a combination of bar plot and polar coordinates with the ggplot2 R package. In this plot, the angles that define each sector are kept fixed but the radii vary proportionally to the square root of the sample size in each subgroup to perceive areas adequately. We colour the areas to encode the information on the treatment effect for each formed subgroup.

In terms of the assessment, the coxcomb plot displays the same information as level plots, therefore satisfying only C1 (effect size), C3 (sample size), and C4 (intersections).
