# Supplementary material for: A critical review of graphics for subgroup analyses in clinical trials
Source: Pharm Stat. 2020 Mar 25;19(5):541–60. doi: 10.1002/pst.2012 (PMC8647927; doi:10.1002/pst.2012)
Supplement: Supplementary file 1 — Appendix S1: Supporting Information [file PST-19-541-s001.zip › PST_2012_pst-18-0123-File002.pdf]

Supplementary Material
Part 1: Additional graphical approaches

for

A Critical Review of Graphics for Subgroup Analyses in Clinical Trials and Some Improvements

Nicolás M. Ballarini[1‡], Yi-Da Chiu[2,3‡], Franz König[1], Martin Posch[1] and Thomas Jaki[4*]

1. Center for Medical Statistics, Informatics, and Intelligent Systems, Medical University of Vienna. Vienna, Austria.
2. Royal Papworth Hospital NHS Foundation Trust. London, U.K.
3. MRC Biostatistics Unit University of Cambridge, School of Clinical Medicine. Cambridge, U.K.
4. Medical and Pharmaceutical Statistics Research Unit, Department of Mathematics and Statistics, Lancaster University. Lancaster, U.K.

‡These authors contributed equally to this work.

* t.jaki@lancaster.ac.uk

A Alternative Galbraith plot

In this alternative the xy-coordinates correspond to the points:

$$x_i = 1/\sqrt{\text{Var}(\hat{\delta}_i - \hat{\delta}_F)}, \quad y_i = (\hat{\delta}_i - \hat{\delta}_F)/\sqrt{\text{Var}(\hat{\delta}_i - \hat{\delta}_F)}$$

where $\hat{\delta}_F$ is the treatment effect estimate in the full population and $\hat{\delta}_i$ is the treatment effect estimate in subgroup i, $i = 1, ..., K$. The central line at $y = 0$ points to the average treatment effect for the full population. The drawback of this modification is that the x-axis does no longer represent the standard error of the treatment effect estimates.

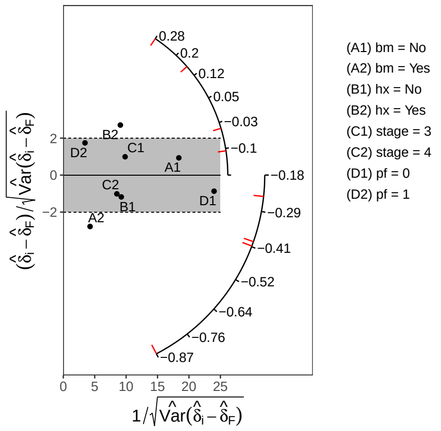

Figure S1.1: Modified Galbraith plot across subgroups defined by stage, history of cardiovascular events (hx) and existence of bone metastasis (bm).

B Alternative STEPP plot with improper axis

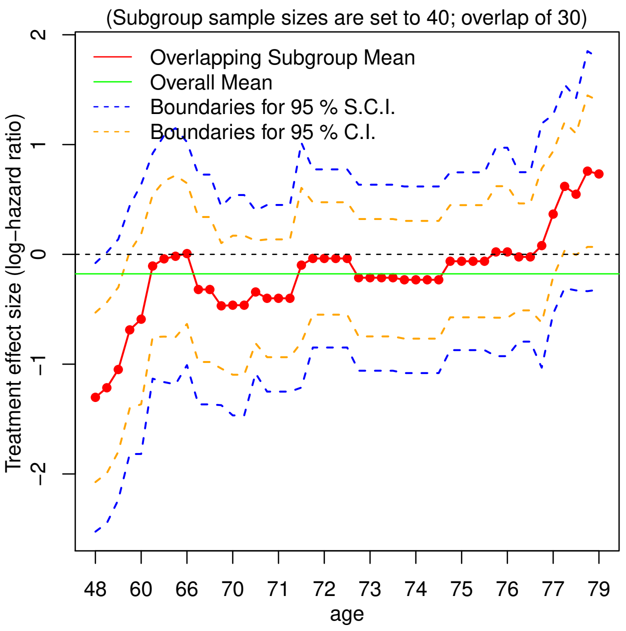

Figure S1.2: STEPP plot of overlapping subgroups defined by age. Each subgroup has a sample size of around $N_{11} = 40$ and is controlled to have about $N_{12} = 30$ subjects overlapping with the neighbouring subgroups.

C Additional graphical approaches

C.1 Graphical approaches with an indirect comparison of treatment effects

In some cases, it may be also interest to visualise responses by treatment arm across subgroups. For example, we may want to display the survival or mortality rate, or simply the mean response if a continuous endpoint is considered. The following plots that we consider are examples of graphics that allow an indirect comparison of treatment effects.

C.1.1 Mosaic Plot

We can use mosaic plots to illustrate event rates per treatment group across the levels of one subgroup-defining covariate, as it is used in[1]. This plot is only appropriate when the endpoint is binary, therefore we use 2-year survival (blue corresponds to 'yes') by treatment and age category (Figure S1.3). In this case, it is possible to observe that the survival rate is larger for treatment in the younger patients while the survival rate is larger for control in the older patients, indicating that the treatment effect may not be homogeneous across the levels of age.

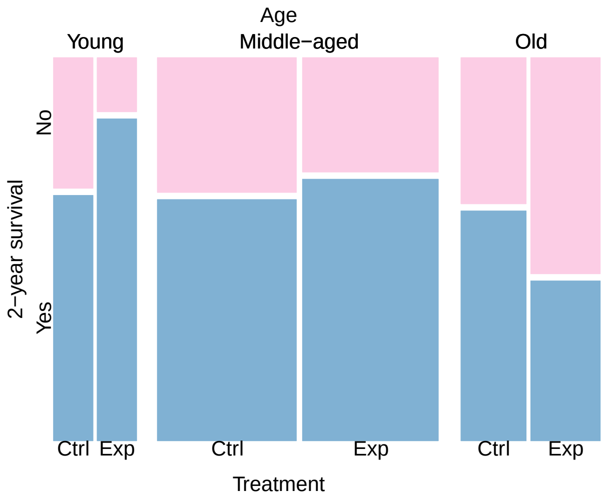

Figure S1.3: Mosaic plot displaying 2-year survival by treatment arm for the subgroups formed by age categories.

C.1.2 Coxcomb plot (Nightingale rose)

In Figure S1.4 we colour the areas according to the 2-year survival rate of each subgroup, while in Figure S1.5, we further divide the plot into treatment and controls arms. This feature allows us to check the sample sizes per subgroup in each treatment arm and may help visualise differences in the survival rates.

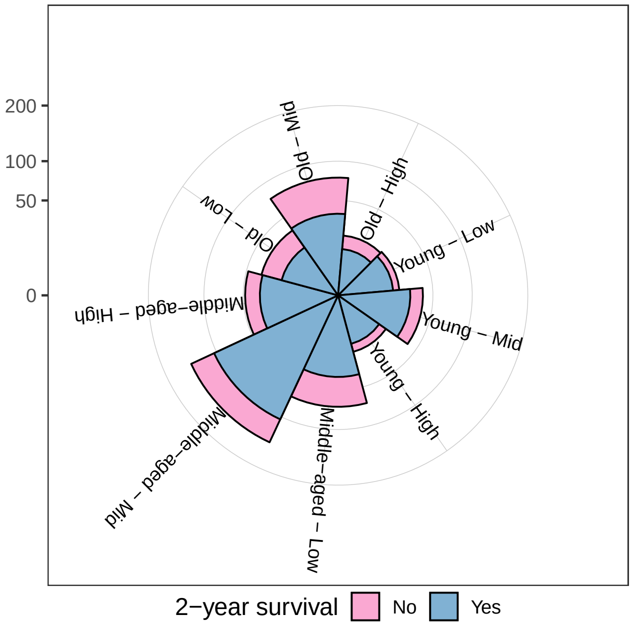

Figure S1.4: Nightingale coxcombs plot for subgroups defined by age and weight with 2-year survival rate. The radius of the sectors are proportional to the square root of the sample sizes in the subgroups.

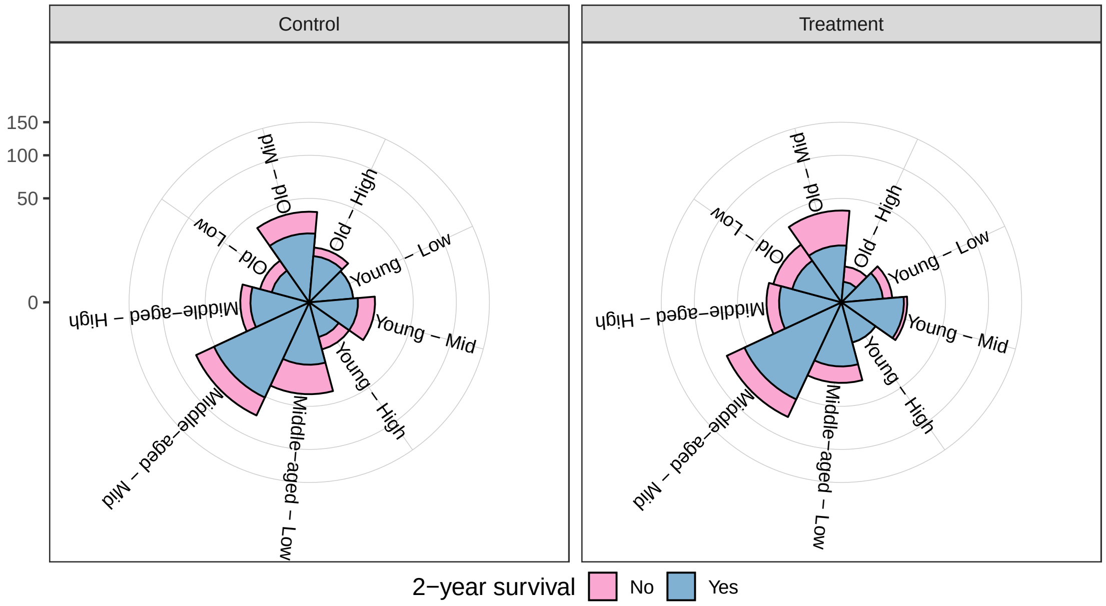

Figure S1.5: Nightingale coxcombs plot for subgroups defined by age and weight with 2-year survival rate and separated by treatment arm. The radius of the sectors are proportional to the square root of the sample sizes in the subgroups.

C.1.3 Alluvial diagram

Alluvial diagrams are flow diagrams that can be used to display the distribution of the subjects across the subgroup-defining covariates. As the mosaic plots, alluvial diagrams may also be used to illustrate event rates per treatment group across the levels of the subgroup-defining covariate.

Figure S1.6 shows one possible implementation of the alluvial plot for the 2-year survival per treatment arm across levels of performance, history of cardiovascular events and bone metastasis. The height of the bars for each category in the subgroup-defining covariates is proportional to the numbers of subjects in this category, therefore giving a notion of the size of the subgroup. Each alluvium (or band) represents the combination of values for the covariates. Therefore this diagram has also the advantage of giving an idea of the overlap of the subgroups, via the width of the bands. The plot is generated using the `alluvial` R package[2].

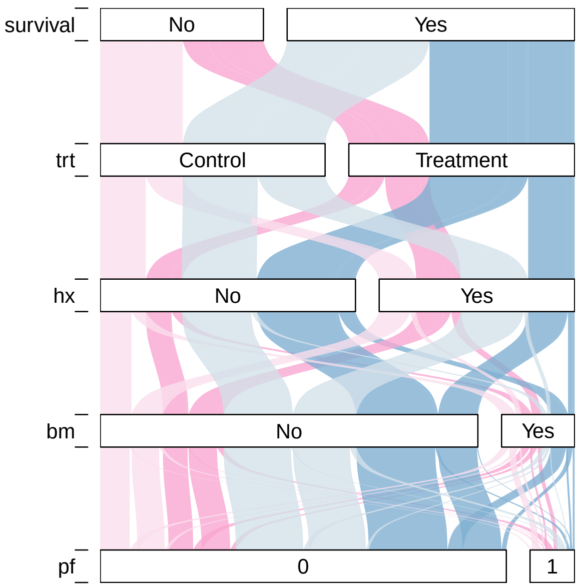

Figure S1.6: Alluvial diagram displaying the distribution of patients across the subgroups defined by history of cardiovascular events (hx), existence of bone metastasis (bm) and performance rating (pf). The dark bands correspond to patients that were randomised to treatment while lighter ones to patients in control. Blue coloured bands represent patients that had survived for at least 2 years, while pink ones represent those who did not. The width of the bands is proportional to the sizes of the subgroups.

C.2 Graphical approaches for subgroup composition

Forest plots, Galbraith plots and L'Abbé plots share the inability of showing subgroup overlaps. One potential improvement is to consider combining relevant figures about overlap information.

Figure S1.7 shows another implementation of alluvial plots for subgroup composition. The blue coloured bands correspond to patients that were randomised to treatment while light-blue bands to patients in control.

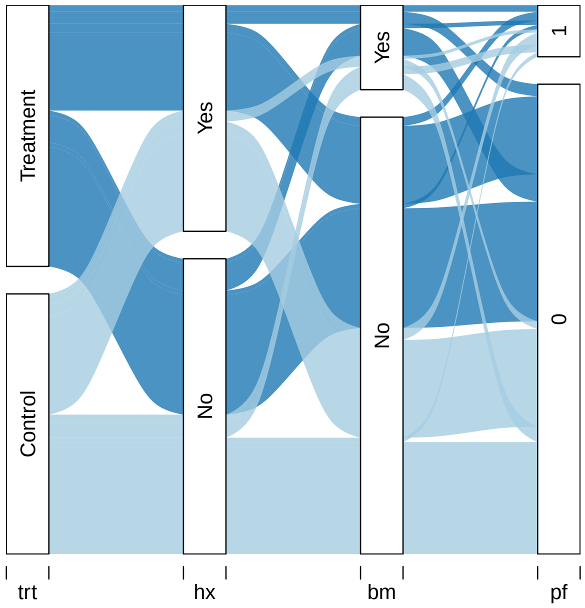

Figure S1.7: Alluvial diagram displaying the distribution of patients across the subgroups defined by history of cardiovascular events (hx), existence of bone metastasis (bm) and performance rating (pf). The dark blue bands correspond to patients that were randomised to treatment while light blue ones to patients in control. The width of the bands is proportional to the sizes of the subgroups.

The plots that are shown in Figure S1.8 exhibit subgroup information about pairwise overlap proportions or similarity measures. Figures S1.8a- S1.8d show pairwise relative overlap proportions, where colours encode the overlap magnitudes. All plots are generated using the `graphics` `R` package, while we also make use of the `diagram` package[3].

More specifically, Figure S1.8a is a plot with bidirectional arrowed curves. The subgroup positioned at the starting point of each arrow is used as a baseline for calculating the relative proportion of the overlapping subgroup. Figure S1.8b is a variant of Figure S1.8a. Two identical sets of subgroup labels around two circles and each shows relative overlap proportions with unidirectional arrowed coloured lines. The subgroup labelled at the starting point of the arrowed line is a baseline subgroup for the relative overlapping proportion. Figure S1.8c is a plot merely using coloured lines connecting subgroup labels on different levels. This plot should be read from top to bottom. A subgroup label on the higher level is the baseline subgroup for the relative overlapping proportions with its counterpart on the lower level. Figure S1.8d is a matrix plot for relative overlapping proportions of pairwise subgroups. The row subgroup label indexes what subgroup should be considered as a baseline and the sizes of the circles signal the overlap magnitude.

Both Figures S1.8e-S1.8f show dissimilarity distance, which is defined by one minus a relative overlap proportion. Each line of Figure S1.8e shows the dissimilarity distance of a subgroup with the others. The red crosses along each line are located according to actual dissimilarity distances; the red subgroup labels correspond to the red crosses, where the labels are placed by order based on their actual dissimilarity distances. Figure S1.8f shows the same information as Figure S1.8e. Note that for each subgroup we do not show its dissimilarity distance to itself and its complement.

Incidentally, the Jaccard index, namely $|A \cap B|/|A \cup B|$ for any sets A, B, can replace the pairwise overlap proportions for subgroup overlap information. The graphical display is thus simplified due to not showing repetitive Jaccard indexes. However, this measure may lead to missing some information about whether a subgroup contains the others or not.

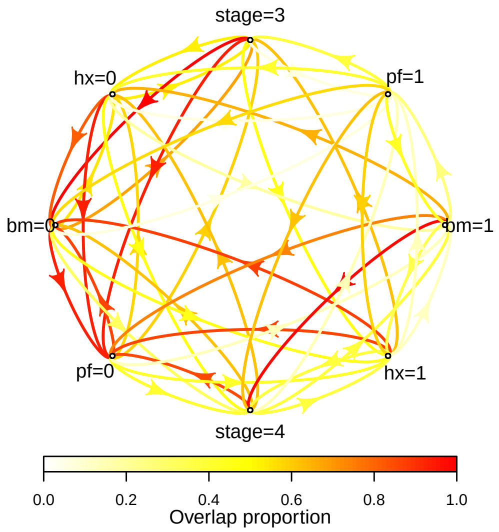

(a) Line plot with bidirectional arrowed curves for relative overlap proportions for pairwise subgroups.

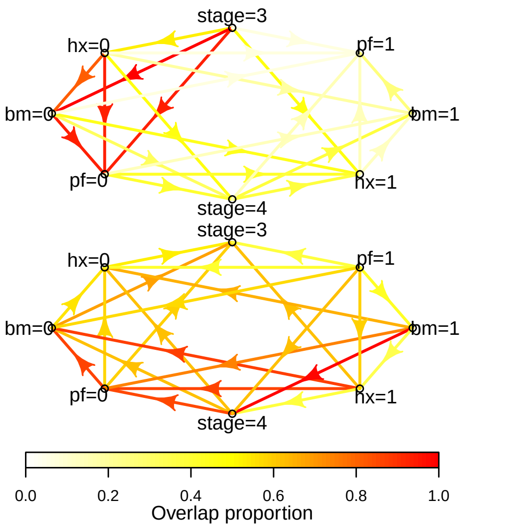

(b) Line plots with unidirectional arrowed lines for relative overlap proportions for pairwise subgroups.

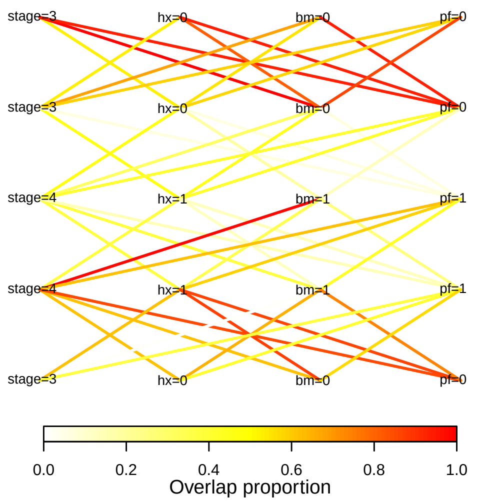

(c) Line plot for relative overlap proportions for pairwise subgroups.

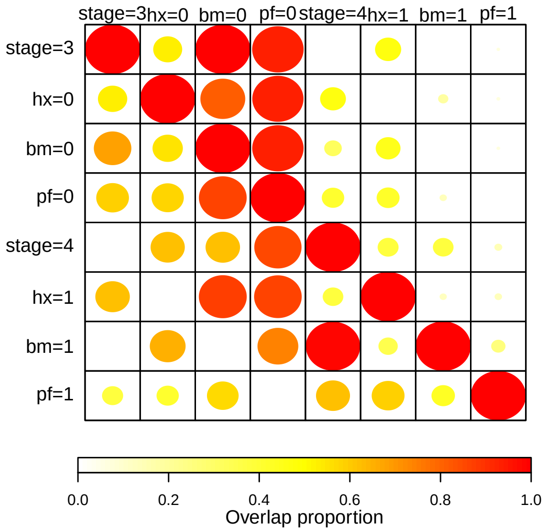

(d) Matrix plot for relative overlap proportions for pairwise subgroups.

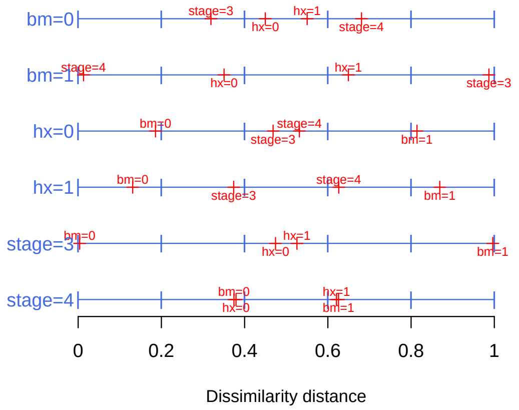

(e) Dissimilarity measures for marginal subgroups.

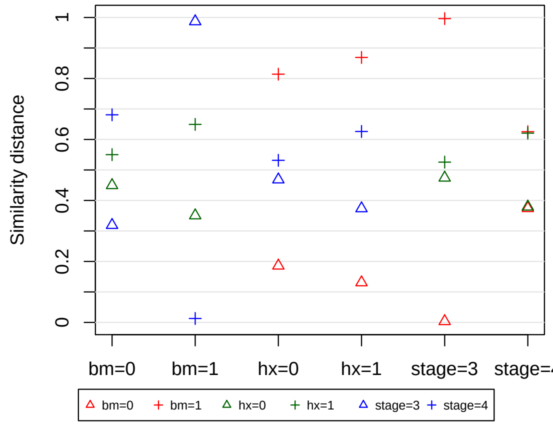

(f) Dot plot for dissimilarity measures for marginal subgroups.

Figure S1.8: Plots for subgroup information about pairwise overlap proportions or dissimilarity measure.

Additional alternatives for the line and chord diagrams display the overlap between the subgroups using a matrix layout (Figures S1.9 - S1.11). These plots may be easier to interpret as they are not overloaded with information and one can focus on one subgroup at the time. However, these plots may be impractical when having a large number of subgroups.

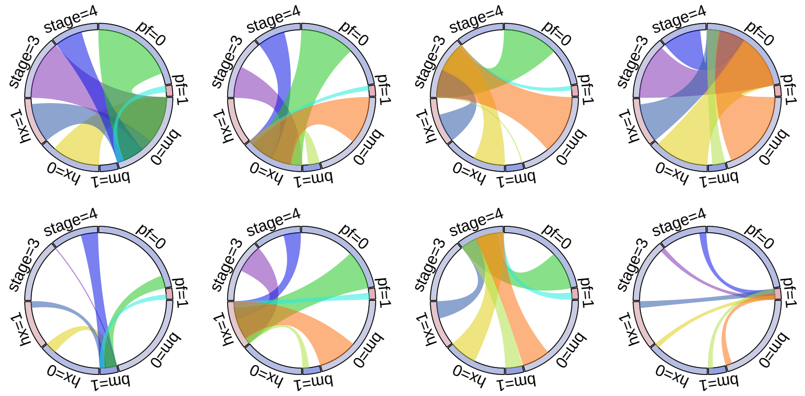

Figure S1.9: Chord diagrams displaying the subgroups formed by the categorised age and weight covariates. The width of each section is proportional to the sample size of the corresponding subgroup. Each circle displays the relative overlap of one subgroup with the others.

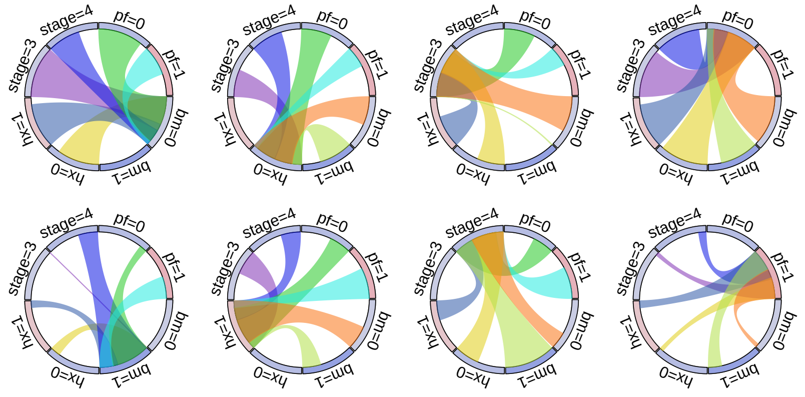

Figure S1.10: Chord diagrams displaying the subgroups formed by the categorised age and weight covariates. Sample sizes are not depicted in this version as the widths are the same for each section. Each circle displays the relative overlap of one subgroup with the others.

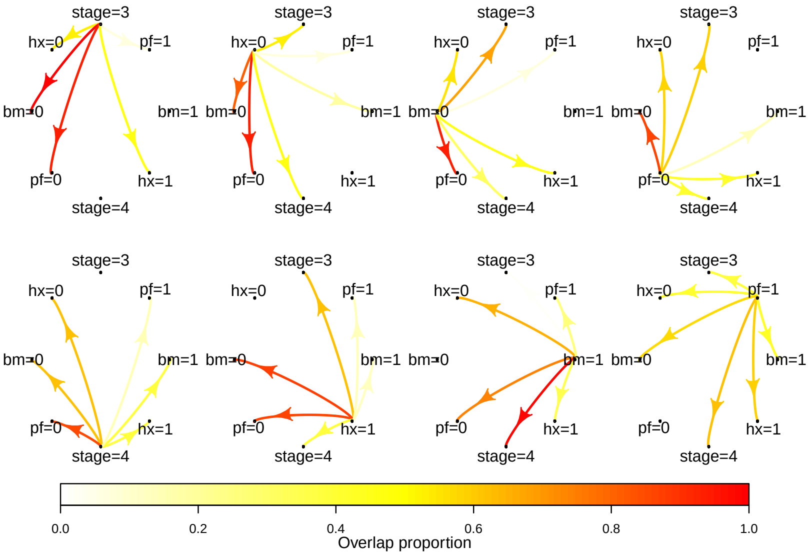

Figure S1.11: Line plots with unidirectional arrowed lines for relative overlap proportions of pairwise subgroups. Each subplot contains the overlap of one subgroup with all the others.

References

1. Heiberger RM, Holland B. Statistical analysis and data display: an intermediate course with examples in R. Springer, 2015.
2. Bojanowski M, Edwards R. alluvial: R Package for Creating Alluvial Diagrams. R package version: 0.1-2. 2016.
3. Soetaert K. diagram: Functions for Visualising Simple Graphs (Networks), Plotting Flow Diagrams. R package version 1.6.4. 2017.
