# Supplementary material for: A critical review of graphics for subgroup analyses in clinical trials
Source: Pharm Stat. 2020 Mar 25;19(5):541–60. doi: 10.1002/pst.2012 (PMC8647927; doi:10.1002/pst.2012)
Supplement: Supplementary file 1 — Appendix S1: Supporting Information [file PST-19-541-s001.zip › PST_2012_pst-18-0123-File003.pdf]

Supplementary Material
Part 2: R code for reproducing the figures in the manuscript

for

A Critical Review of Graphics for Subgroup Analyses in Clinical Trials and Some Improvements

Nicolás M. Ballarini, Yi-Da Chiu, Franz Koenig, Martin Posch and Thomas Jaki

Contents

1 Overview **2**

Installation **2**

Getting started **2**

2 Graphics in the manuscript **3**
2.1 Forest Plot . . . 3
2.2 Upset plot . . . 4
2.3 Galbraith plot . . . 6
2.4 STEPP . . . 7
2.5 Contour plot . . . 8

3 Graphics in the appendix **9**
3.1 Tree plot . . . 9
3.2 Level Plot . . . 10
3.3 Mosaic Plot . . . 12
3.4 Venn diagram . . . 13
3.5 Bar chart . . . 15
3.6 L'Abbé plot . . . 16
3.7 Chord diagram . . . 17
3.8 Nightingale Rose . . . 18

4 Graphics in the supplementary material **19**
4.1 Alternative Galbraith plot . . . 19
4.2 Alternative STEPP plot with improper axis . . . 20
4.3 Graphical approaches with an indirect comparison of treatment effects . . . 21
4.3.1 Mosaic Plot . . . 21
4.3.2 Nightingale Rose . . . 22
4.3.3 Alluvial Plot . . . 24
4.4 Graphical approaches for subgroup composition . . . 25
4.4.1 Pairwise overlap . . . 25
4.4.2 Chord diagrams in matrix layout . . . 27

5 R Session Info **30**

1 Overview

In this vignette we showcase all plots in the manuscript with their corresponding code to illustrate the usage of the `SubgrPlots` package.

Installation

The package can be installed directly from CRAN:

```
install.packages("SubgrPlots")
```

or the development version from github:

```
## For dev version  needs devtools package
# install.packages("devtools")
devtools::install_github("nicoballarini/SubgrPlots")
```

Getting started

We use the `prca` dataset that is available in the package. The dataset is also available in Royston and Sauerbrei (2018)[1].

```
# Loads the SubgrPlots package available as supplementary material from the
# manuscript website. Install it first!!
library(SubgrPlots)
#> Warning: package 'SubgrPlots' was built under R version 3.5.2
library(dplyr) # For some data wrangling
#>
#> Attaching package: 'dplyr'
#> The following objects are masked from 'package:stats':
#>
#>     filter, lag
#> The following objects are masked from 'package:base':
#>
#>     intersect, setdiff, setequal, union
# Load the dataset to be used
data(prca)
head(prca)
#>   survtime cens rx bm hx stage pf age weight age1 age2 wt1 wt2 age_group
#> 1     72.5    0  0  0  0     3  0  75     76    1    0   0   0   (65,75]
#> 2     40.5    1  1  0  1     3  0  69    102    1    0   1   0   (65,75]
#> 3     20.5    1  0  0  1     3  1  75     94    1    0   1   0   (65,75]
#> 4     65.5    0  0  0  0     3  0  67     99    1    0   1   0   (65,75]
#> 5     24.5    1  0  0  0     3  0  71     98    1    0   1   0   (65,75]
#> 6     46.5    1  0  0  0     3  0  75    100    1    0   1   0   (65,75]
#>   weight_group
#> 1      [69,90]
#> 2     (90,110]
#> 3     (90,110]
#> 4     (90,110]
#> 5     (90,110]
#> 6     (90,110]
```

[1] Royston P, Sauerbrei W. Multivariable model-building: a pragmatic approach to regression anaylsis based on fractional polynomials for modelling continuous variables. John Wiley & Sons; 2008 Sep 15. (https://www.imbi.uni-freiburg.de/Royston-Sauerbrei-book/index.html#datasets - accessed 15th December 2018)

2 Graphics in the manuscript

2.1 Forest Plot

```
dat = prca
dat = dat %>%
  mutate(bm = factor(ifelse(bm == 0 , "No", "Yes")),
         hx = factor(ifelse(hx == 0 , "No", "Yes")))

## Figure 1. Forest Plot -----------------------------------------------------
main.title = list("", "Forest plot of subgroups",
                  "Kaplan-Meier curves\n by treatment group")
label.x = list("", "Log hazard ratio",
               "Time (days)")
plot_forest(dat,
            covari.sel = c(4, 5, 6, 7),
            trt.sel = 3,
            resp.sel = c(1, 2),
            outcome.type = "survival",
            size.shape = c(0.3, 6.5 / 4),
            font.size = c(1, 1, 1, .8),
            title = main.title,
            lab.x = label.x, time = 50, KM = TRUE,
            show.km.axis = TRUE,
            n.brk = 6, max.time = 70,
            widths = c(2, 1.5, 1))
```

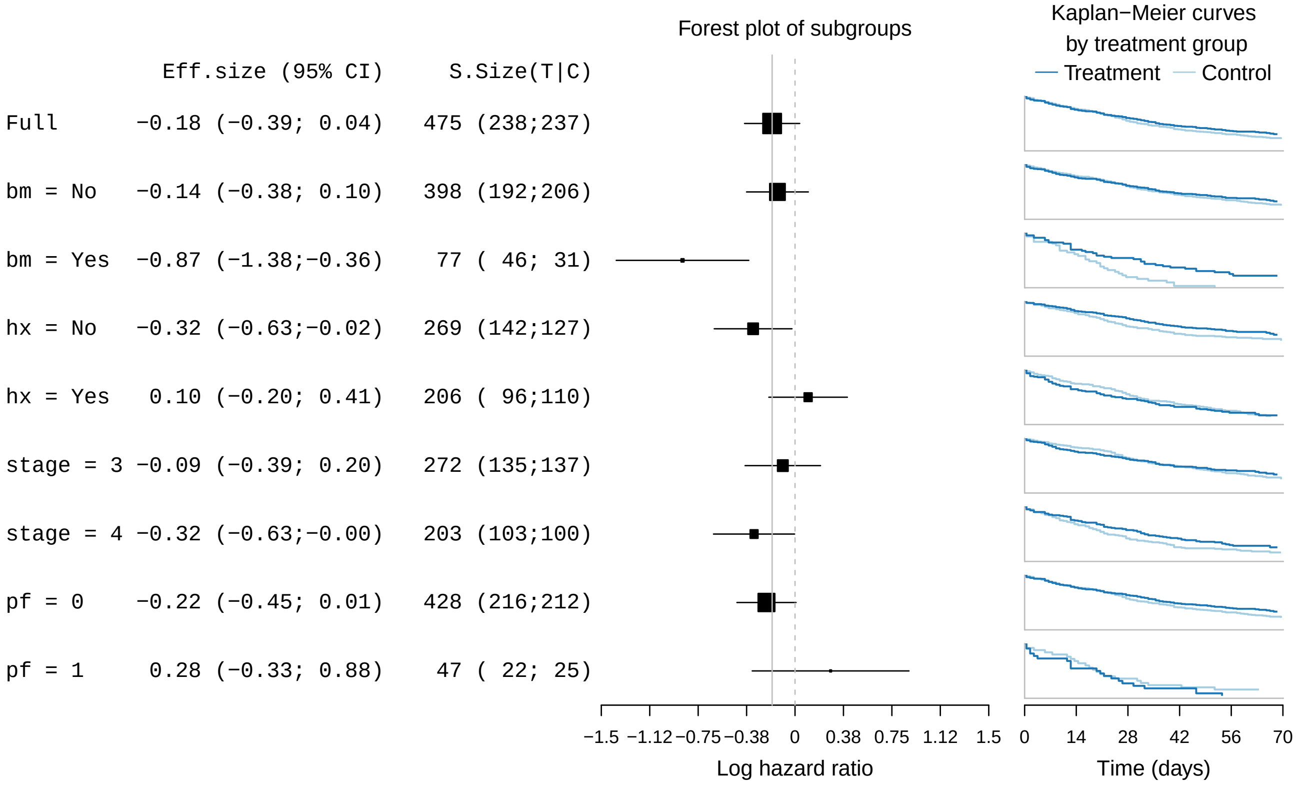

Figure S2.1: Forest plot for subgroups defined by performance (pf), stage, history of cardiovascular events (hx) and existence of bone metastasis (bm). Effect sizes in terms of the log-hazard ratio and associated treatment and control group Kaplan-Meier curves are displayed.

2.2 Upset plot

```r
library(UpSetR) # We use the UpSetR package for drawing the original upset plot
dat = prca
# We need the dataset in the upset format.
# First variable is treatment to be a labelled factor
# Then all subgroup defining covariates.
# And finally, the response variable
prca.upset = data.frame(trt = factor(ifelse(prca$rx == 1, "Experimental", "Control")),
                        bm = 1 * (prca$bm == 1),
                        pf = 1 * (prca$pf == 1),
                        hx = 1 * (prca$hx == 1),
                        stage = 1 * (prca$stage == 4),
                        survtime = prca$survtime,
                        cens = prca$cens == 1)

## Figure 2. UpSet -------------------------------------------------------------
# Create a custom query to operate on the rows of the data and display colors by treatments
Myfunc = function(row, param1, param2) {
  data = (row["trt"] %in% c(param1, param2))
}
pal = c("#1f78b4", "#a6cee3")
upset(prca.upset,
      order.by = "freq",
      sets = c("bm",'pf',"hx",'stage'),
      nintersects = 14,
      text.scale = 1.4,
      queries = list(list(query = Myfunc,
                          params = c("Control", "Experimental"),
                          color = pal[2],
                          active = T,
                          query.name = "Control"),
                     list(query = Myfunc,
                          params = c("Experimental", "Experimental"),
                          color = pal[1],
                          active = T,
                          query.name = "Experimental")),
      query.legend = "top")
```

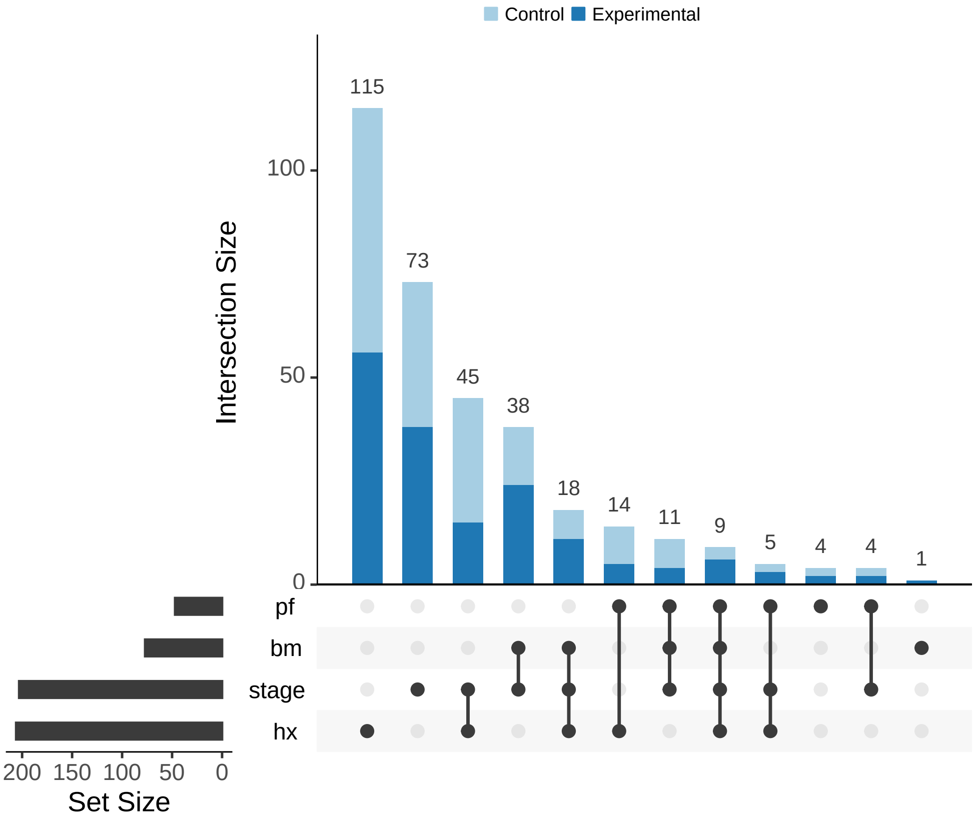

Figure S2.2: Upset plot displaying the subgroups formed by the intersection of all subgroup-defining covariates.

```
dat = prca
## Figure 3. SubgroUpSet -----------------------------------------------------
# We need the dataset in the upset format.
# First variable is treatment to be a labelled factor
# Then all subgroup defining covariates.
# And finally, the response variable
dat = data.frame(trt = factor(ifelse(prca$rx == 1, "Experimental", "Control")),
                 bm = 1 * (prca$bm == 1),
                 pf = 1 * (prca$pf == 1),
                 hx = 1 * (prca$hx == 1),
                 survtime = prca$survtime,
                 cens = 1 * (prca$cens == 1))
# We now used the function `subgroupset` from the SubgrPlots package
# to display treatment effects
subgroupset(dat,
            order.by = "freq",
            empty.intersections = "on",
            sets = c("bm", 'pf', "hx"),
            text.scale = 1.5,
            mb.ratio = c(0.25, 0.45, 0.30),
            treatment.var = "trt",
            outcome.type = "survival",
            effects.summary = c("survtime", "cens"),
            query.legend = "top", icon = "pm", transpose = TRUE)
```

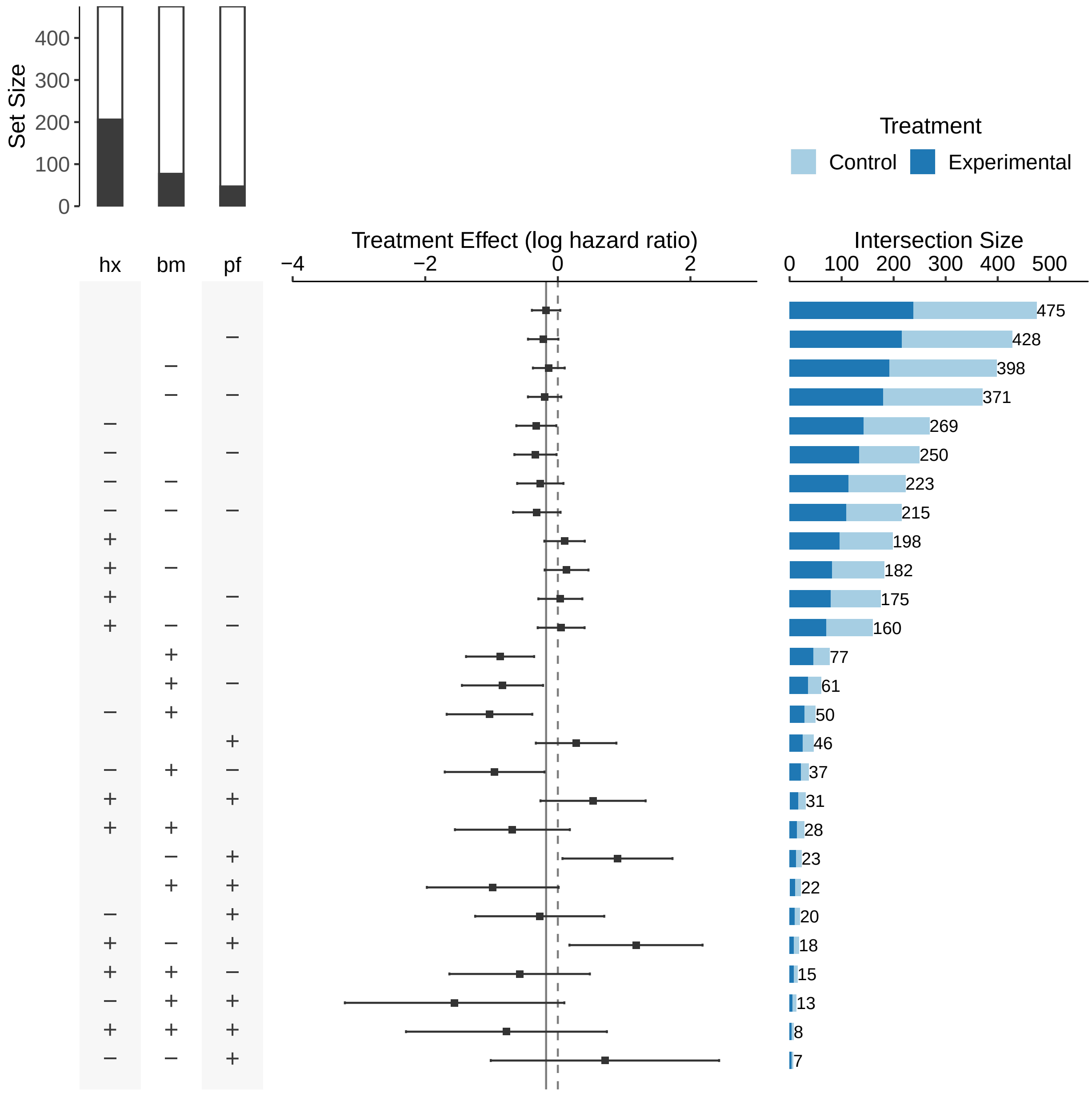

Figure S2.3: Improved UpSet plot for subgroups defined by performance (pf), bone metastasis (bm) and history of cardiovascular events (hx). The panel on the left (matrix) displays how the subgroups are formed by assigning a '+' if the variable is equal to 1 and a '-' if the variable is equal to 0. The bar plot on top of the matrix panel indicates the marginal set sizes in relation to the total sample size, with the black region corresponding to the 1 or 'yes' category and the white region corresponding to the 0 or 'no' category. Treatment effect sizes and their confidence intervals are displayed in the panel on the middle and the subgroup sizes in the horizontal bar plot on the right.

2.3 Galbraith plot

```
dat = prca
dat = dat %>%
  mutate(bm = factor(ifelse(bm == 0 , "No", "Yes")),
         hx = factor(ifelse(hx == 0 , "No", "Yes")))
## Figure 4. Galbraith plot ----------------------------------------------------
p = ggplot_radial(dat,
                  covari.sel = c(4, 5, 6, 7),
```

```
                    trt.sel = 3,
                    resp.sel = c(1, 2),
                    outcome.type = "survival",
                    range.v = c(-8, 6),
                    font.size = 4,
                    lab.xy = "default",
                    ticks.length = 0.05)
p +
  ggplot2::theme(text = ggplot2::element_text(size = 14))
```

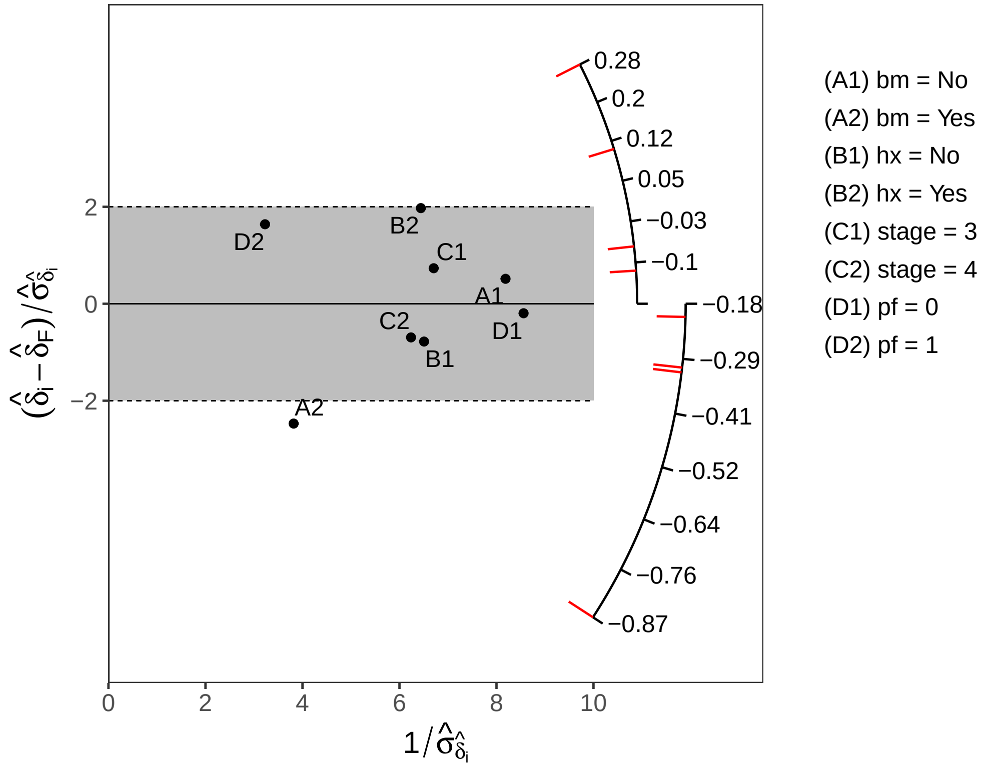

Figure S2.4: Galbraith plot for subgroups defined by existence of bone metastasis (bm), history of cardiovascular events (hx), stage, and performance rating (pf).

2.4 STEPP

```
dat = prca

## Figure 5. STEPP Plot ------------------------------------------------------------
lab.y.title = paste("Treatment effect size (log-hazard ratio)");
setup.ss = c(30, 40)
sub.title = paste0("(Subgroup sample sizes are set to ", setup.ss[2], "; overlap of ", setup.ss[1], ")")
p = ggplot_stepp(dat,
           covari.sel = 8,
           trt.sel = 3,
           resp.sel = c(1, 2),
           outcome.type = "survival",
           setup.ss = setup.ss,
           alpha = 0.05,
           title = NULL,
           lab.y = lab.y.title,
           subtitle = sub.title)
```

```
#> The subgroup sample sizes are actually 46 44 43 44 42 56 48 48 61 53 53 75 64 64 111 83 83 83 47 87 87
p  +
  ggplot2::theme(text = ggplot2::element_text(size = 14))
```

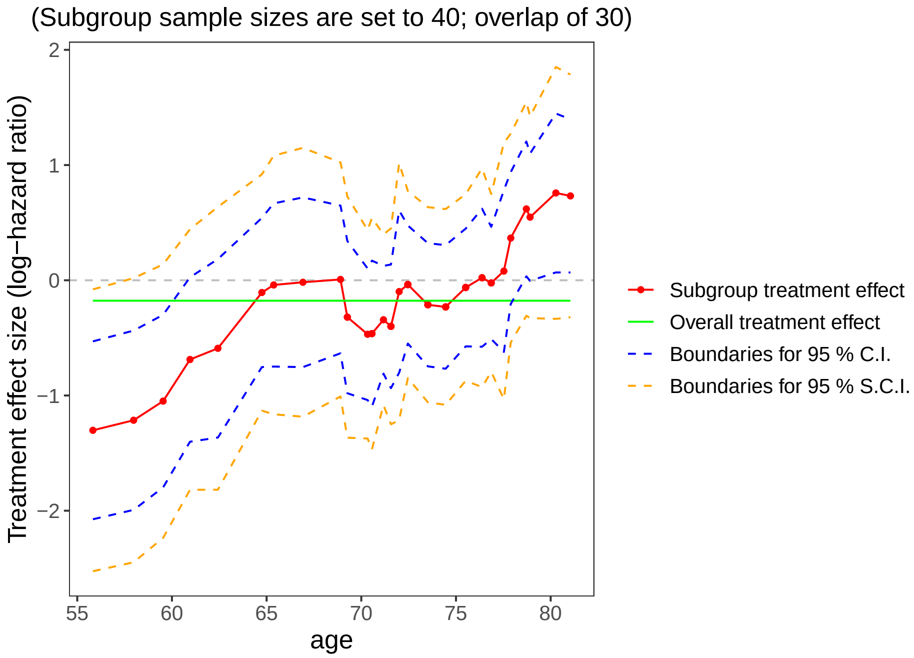

Figure S2.5: STEPP plot of overlapping subgroups defined by age. Each subgroup has a sample size of around $N_{11} = 40$ and is controlled to have about $N_{11} = 30$ subjects overlapping with the neighbouring subgroups.

2.5 Contour plot

```
dat = prca
setup.ss =  c(10, 60, 15, 30)
dat = dat %>%
  rename(Weight = weight,
         Age = age)

## Figure 6. Contour plot with sliding windows --------------------------------
plot_contour(dat,
             covari.sel = c(8, 9),
             trt.sel = 3,
             resp.sel = c(1, 2),
             outcome.type = "survival",
             setup.ss =  setup.ss,
             n.grid = c(100, 100),
             brk.es = seq(-4.5, 4.5, length.out = 101),
             n.brk.axis =  7,
             para.plot = c(0.5, 2, 6),
             font.size = c(1, 1, 1, 1, 1),
             title = NULL,
             strip = paste("Treatment effect size (log hazard ratio)"),
             show.overall = T, show.points = T,
             filled = T, palette = "hcl", col.power = 0.75)
#> The number of subgroups over the first covariate is 10
#> The subgroup sample sizes over the first covariate are actually 62 67 89 83 87 119 117 87 81 29
#> The number of further divided subgroups over the second covariate is 4 4 5 5 5 7 7 5 5 1
#> Overall Treatment effect is: -0.1774, with confidence interval: (-0.3912;0.0365)
```

```r
## Contour plot with weighted local regression --------------------------------
plot_contour_localreg(dat,
                      covari.sel = c(8, 9),
                      trt.sel = 3,
                      resp.sel = c(1, 2),
                      n.grid = c(100, 100),
                      font.size = c(1, 1, 1, 1, 1),
                      brk.es = seq(-4.5, 4.5, length.out = 101),
                      n.brk.axis =  7,
                      strip = "Treatment effect size (log hazard ratio)",
                      outcome.type = "survival")
#> Overall Treatment effect is: -0.1774, with confidence interval: (-0.3912;0.0365)
```

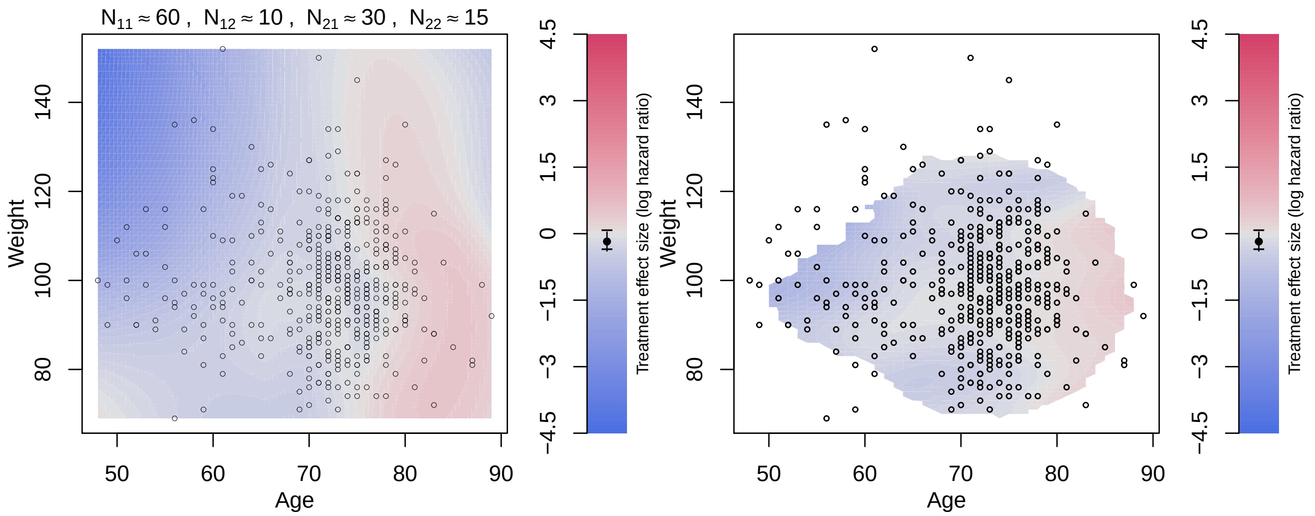

Figure S2.6: Contour plot of treatment effect in terms of the log-hazard ratio over the plane of age and weight. (a) Contour lines are drawn by forming subgroups with neighbouring subjects, calculating the treatment effect for subgroups and interpolating the results using loess. N_{11} stands for the sample size of a marginal subgroup defined by a range of age, N_{12} is the overlap size of the immediate marginal subgroups on age, N_{21} is the sample size of the subset of a marginal subgroup on age but further defined by a range of weight, and N_{22} is the overlap size of the immediate subgroups (which are the subset of a marginal subgroup on age) on weight. (b) Contour lines are drawn by fitting a local regression at each point of the grid, using subjects weights according to their distance to the point of the grid. Points with few subjects in the vicinity of the grid point were left blank.

3 Graphics in the appendix

3.1 Tree plot

```r
dat = prca
dat = dat %>%
  mutate(bm = factor(ifelse(bm == 0 , "No", "Yes")),
         hx = factor(ifelse(hx == 0 , "No", "Yes")))


# Figure A.1. Tree Plot
# Tree plot with y-axis separately for each layer ------------------------------
plot_tree(dat,
          covari.sel = c(4, 5, 7),
          trt.sel = 3,
          resp.sel = c(1, 2),
          outcome.type = "survival",
```

```
          add.aux.line = TRUE,
          font.size = c(12, 12, 0.8),
          title = NULL, text.shift = 0.01,
          lab.y = "Effect size (log hazard ratio)",
          keep.y.axis = FALSE)

# Tree plot with consistent y-axis across layers ---------------------
plot_tree(dat,
          covari.sel = c(4, 5, 7),
          trt.sel = 3,
          resp.sel = c(1, 2),
          outcome.type = "survival",
          add.aux.line = TRUE,
          font.size = c(12, 12, 0.8),
          text.shift = 0.01,
          title = NULL,
          lab.y = "Effect size (log hazard ratio)",
          keep.y.axis = TRUE)
```

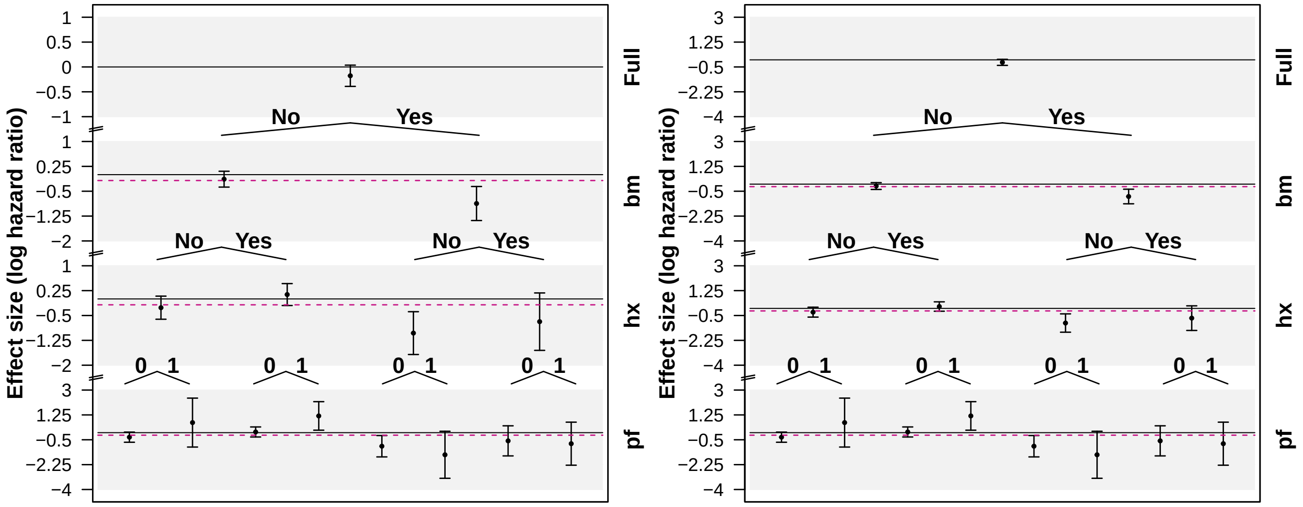

Figure S2.7: Tree plot for the treatment effect in terms of the log-hazard ratio for subgroups defined by category combinations of existence of bone metastasis (bm), history of car- diovascular events (hx), and performance rating (pf). Each layer shows the 95% C.I. of treatment effect differences for the associated subgroups. The purple horizontal lines placed in the middle of the C.I. have a length proportional to the weight of subgroup sample size over the full population. In (a) the y-axes are independent in each layer of the plot, while in (b) y-axes are kept fixed across levels, which allows comparing variability in the estimates.

3.2 Level Plot

```
dat =  prca
levels(dat$age_group)    = c("Young", "Middle-aged", "Old")
levels(dat$weight_group) = c("Low", "Mid", "High")
names(dat)[c(14, 15)] = c("Age", "Weight")
strip.title = "Treatment effect size (log hazard ratio)"

## Figure A.2 Level plot -------------------------------------------------------
plot_level(dat,
           covari.sel = c(14, 15),
           trt.sel = 3,
           resp.sel = c(1, 2),
           outcome.type = "survival",
```

```
           ss.rect = FALSE,
           range.strip = c(-3, 3),
           n.brk = 31,
           n.brk.axis =  7,
           font.size = c(14, 12, 1, 14, 1),
           title = paste0("Total sample size = ", nrow(dat)),
           strip = strip.title, effect = "HR",
           show.overall = TRUE, palette = "hcl")
#> The minimum of treatment effect sizes is -2.426052
#> The maximum of treatment effect sizes is 0.3349348
#> Overall Treatment effect is: -0.1774, with confidence interval: (-0.3912;0.0365)

## Cells proportional to sample sizes ----------------------------------------
plot_level(dat,
           covari.sel = c(14, 15),
           trt.sel = 3,
           resp.sel = c(1, 2),
           outcome.type = "survival",
           ss.rect = TRUE,
           range.strip = c(-3, 3),
           n.brk = 31,
           n.brk.axis =  7,
           font.size = c(14, 12, 1, 14, 1),
           title = paste0("Total sample size = ", nrow(dat)),
           strip = strip.title, show.overall = TRUE, palette = "hcl")
#> The minimum of treatment effect sizes is -2.426052
#> The maximum of treatment effect sizes is 0.3349348
#> Overall Treatment effect is: -0.1774, with confidence interval: (-0.3912;0.0365)
```

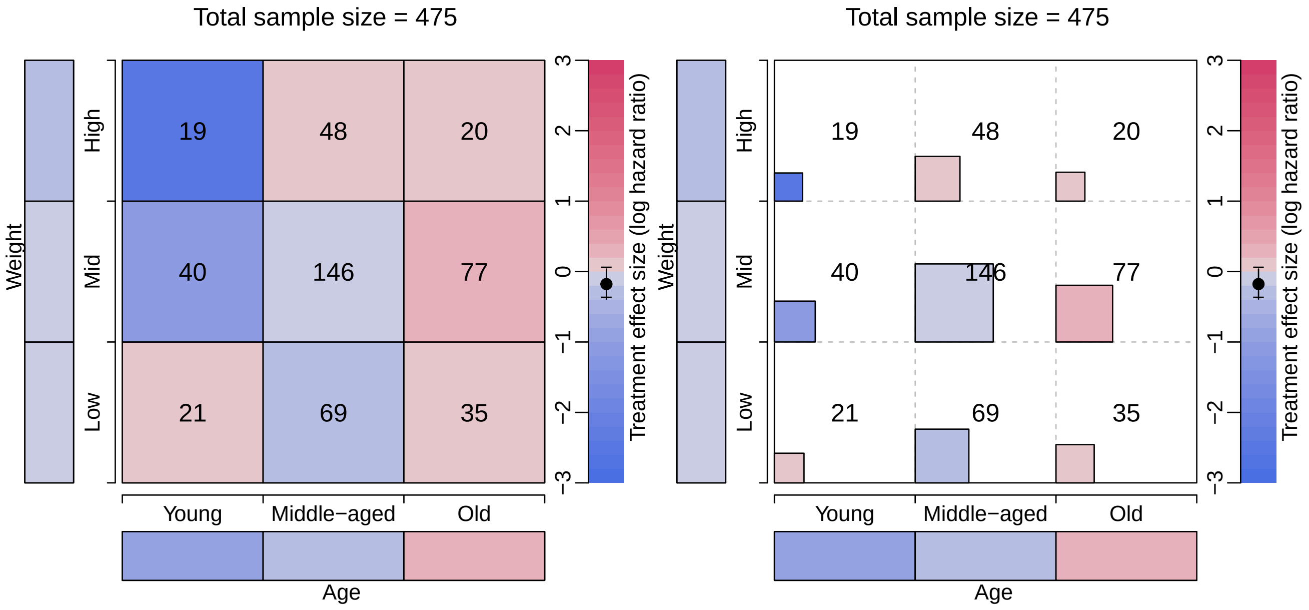

Figure S2.8: Level plots of treatment effect in terms of the log-hazard ratio across mutually disjoint subgroups defined by age and weight categorised in three levels. The cells on the bottom and the left margins correspond to the marginal subgroups defined by the levels of age and weight. In (b) the area of each square inside the cells is proportional to the sample sizes, which are also displayed in the middle of the cells.

3.3 Mosaic Plot

```
dat = prca
levels(dat$age_group) = c("Young", "Middle-aged","Old")
levels(dat$weight_group)  = c("Low", "Mid", "High")
# Change variable names for better presentation
dat = dat %>%
  rename(`Bone Metastasis` = bm,
         `Performance rating` = pf,
         `History of cardiovascular events` = hx,
         Weight = weight_group,
         Age = age_group)

# Figure A.3 Mosaic plot with two covariates ------------------------------------
plot_mosaic(dat = dat,
            covari.sel = c(14, 15),
            trt.sel = 3,
            resp.sel = c(1, 2),
            outcome.type =  "survival",
            range.v = NULL,
            adj.ann.subgrp = 4,
            range.strip = c(-3, 3),
            n.brk = 31,
            n.brk.axis = 7, sep. = 0.034,
            font.size = c(10, 12, 12, 10, 1),
            title = NULL, lab.xy = NULL,
            strip = "Treatment effect size (log-hazard ratio)",
            col.line = "white", lwd. = 2,
            effect = "HR", print.ss = FALSE, palette = "hcl")
#> The minimum of treatment effect sizes is -2.426052
#> The maximum of treatment effect sizes is 0.3349348
#> Overall Treatment effect is: -0.1774, with confidence interval: (-0.3912;0.0365)
# Mosaic plot with three covariates --------------------------------------------
plot_mosaic(dat = dat,
            covari.sel = c(5, 7, 4),
            trt.sel = 3,
            resp.sel = c(1, 2),
            outcome.type =  "survival",
            range.v = NULL, adj.ann.subgrp = 4,
            range.strip = c(-3, 3),
            n.brk = 31, n.brk.axis = 7,
            font.size = c(12, 12, 12, 10, 1),
            title = NULL, lab.xy = NULL,
            strip = "Treatment effect size (log-hazard ratio)",
            effect = "HR", palette = "hcl")
#> The minimum of treatment effect sizes is -1.557225
#> The maximum of treatment effect sizes is 1.178853
#> Overall Treatment effect is: -0.1774, with confidence interval: (-0.3912;0.0365)
```

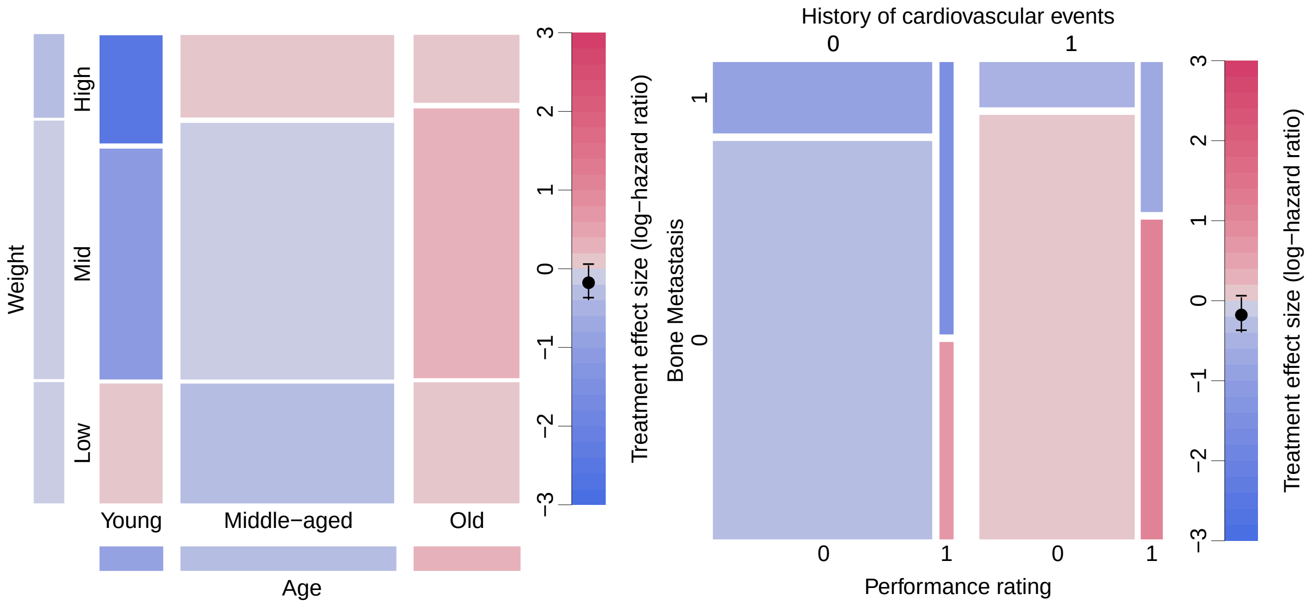

Figure S2.9: (a) Mosaic plot of treatment effect in terms of the log-hazard ratio across mutually disjoint subgroups defined by age and weight categorised in three levels. The cells on the bottom and the left margins correspond to the marginal subgroups defined by the levels of age and weight. The area of each mosaic is proportional to the sample sizes. (b) Mosaic plot of treatment effect in terms of the log-hazard ratio across mutually disjoint subgroups defined by history of cardiovascular events, performance rating and bone metastasis.

3.4 Venn diagram

```
dat = prca
dat = dat %>%
  rename(Performance = pf,
         `Bone\nmetastasis` = bm,
         `History of\ncardiovascular\nevents` = hx)

## Figure A.4. Venn Diagram with three covariates -------------------------------
plot_venn(dat,
          covari.sel = c(5, 7, 4),
          cat.sel   = c(2, 2, 2),
          trt.sel   = 3,
          resp.sel = c(1, 2),
          outcome.type = "survival",
          fill      = FALSE,
          cat.dist  = c(0.03, 0.04, 0.08),
          font.size = c(1, 1.29, 1.4, 1, 1, 1))
#> The minimum of treatment effect sizes is -1.557225
#> The maximum of treatment effect sizes is 1.178853
```

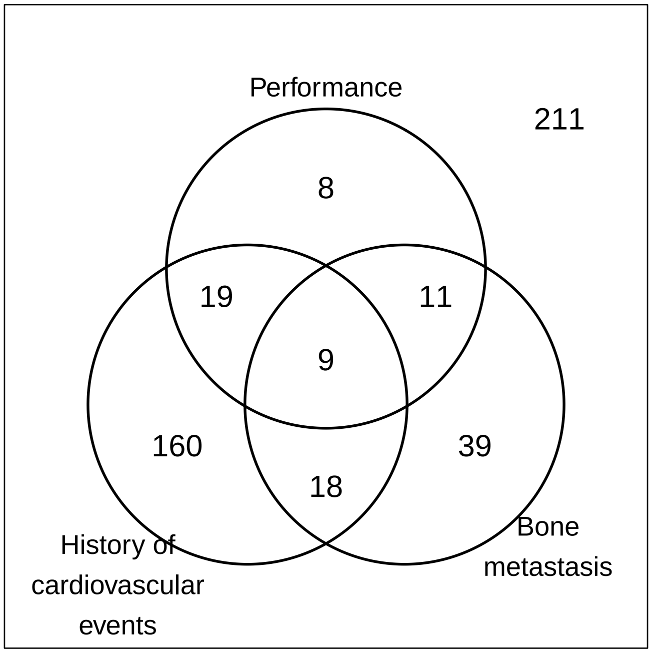

Figure S2.10: (a) Venn diagram of 3 subgroups defined by presence of bone metastasis, history of cardiovascular events, and performance rating = 1.

```
dat = prca
dat = dat %>%
  rename(Stage = stage,
         Performance = pf,
         `Bone\nmetastasis` = bm,
         `History of\ncardiovascular\nevents` = hx)
## Venn Diagram with four covariates ------------------------------------------
plot_venn(dat,
          covari.sel = c(4, 6, 7, 5),
          cat.sel = c(2, 2, 2, 2),
          trt.sel = 3,
          resp.sel = c(1, 2),
          outcome.type = "survival",
          fill = TRUE,
          range.strip = c(-3, 3),
          n.brk = 31, n.brk.axis = 7,
          font.size = c(0.5, 1.1, 1.4, 1, 1, 1),
          strip = paste("Treatment effect size (log hazard ratio)"),
          palette = "hcl",
          cat.dist = c(0.22, 0.22, 0.11, 0.16))
#> The minimum of treatment effect sizes is -1.557225
#> The maximum of treatment effect sizes is 1.010984
#> Overall Treatment effect is: -0.1774, with confidence interval: (-0.3912;0.0365)

#------------------------------------------------------------------------------
dat =  prca
dat = dat %>%
  rename(Stage = stage,
         Performance = pf,
         `Bone\nmetastasis` = bm,
         `History of\ncardiovascular events` = hx)
## Venn Diagram with three covariates (proportional area) ---------------------
plot_venn(dat,
```

```
            covari.sel = c(5, 7, 4),
            cat.sel = c(2, 2, 2),
            trt.sel = 3,
            resp.sel = c(1, 2),
            outcome.type = "survival",
            fill = TRUE,
            range.strip = c(-3, 3),
            n.brk = 31, n.brk.axis = 7,
            font.size = c(1, 1.29, 1.4, 1, 1, 1),
            strip = paste("Treatment effect size (log hazard ratio)"),
            palette = "hcl", prop_area = TRUE)
#> The minimum of treatment effect sizes is -1.557225
#> The maximum of treatment effect sizes is 1.178853
#> Overall Treatment effect is: -0.1774, with confidence interval: (-0.3912;0.0365)
```

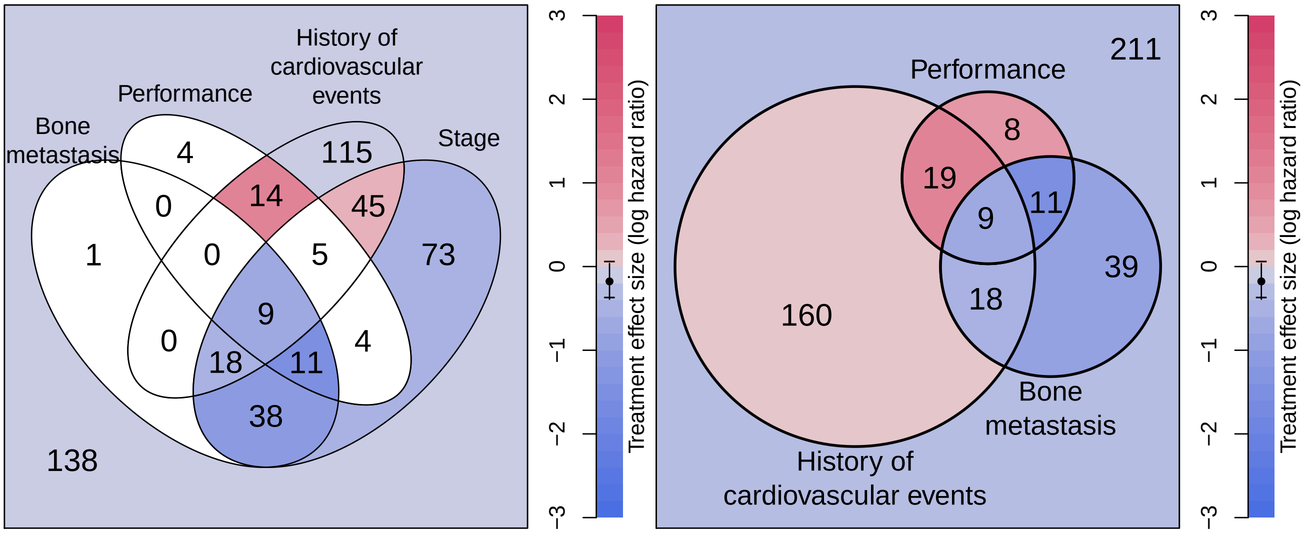

Figure S2.11: (b) Venn diagram of 4 sets defined by presence of bone metastasis, disease stage, performance rating = 1 and history of cardiovascular events with treatment effect sizes in terms of the log-hazard ratios. (c) Approximate area-proportional Venn diagram of 3 subgroups defined by presence bone metastasis, history of cardiovascular events and performance rating = 1 with treatment effect sizes in terms of the log-hazard ratios.

3.5 Bar chart

```
dat = prca
levels(dat$age_group)     = c("Young", "Middle-aged", "Old")
levels(dat$weight_group)  = c("Low", "Mid", "High")
names(dat)[c(14, 15)]      = c("Age", "Weight")

## Figure A.5. Bar chart --------------------------------------------------------
plot_barchart(dat,
              covari.sel = c(14, 15),
              trt.sel = 3,
              resp.sel = c(1, 2),
              outcome.type = "survival",
              font.size = c(12, 12, 12, 1), time = 50,
              lab.y = "Treatment effect size (RMST difference)")
```

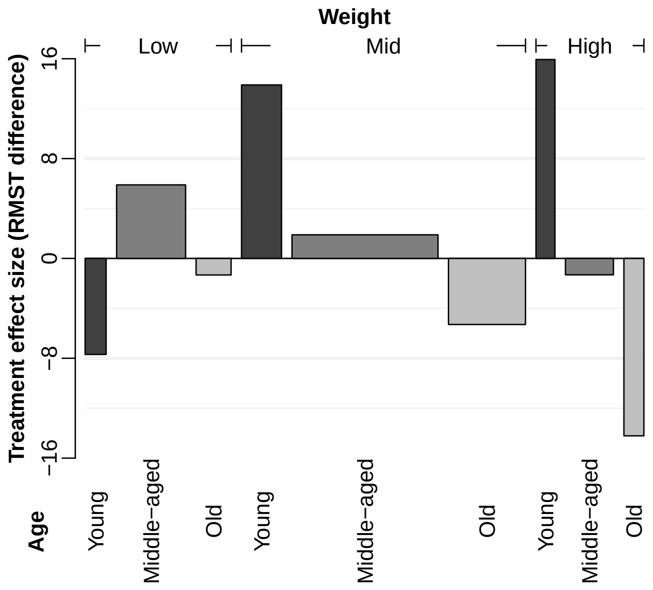

Figure S2.12: Bar plot of treatment effect in terms of the difference in restricted mean survival time across mutually disjoint subgroups defined by age and weight categorised in three levels. The width of each bar is proportional to the sample size for subgroups. The area can be interpreted as the gain/loss in restricted mean survival when using treatment in comparison to control. The black, grey and light grey indicate the age categories young, middle-aged and old, respectively.

3.6 L'Abbé plot

```
dat = prca
dat = dat %>%
  mutate(bm = factor(ifelse(bm == 0 , "No", "Yes")),
         hx = factor(ifelse(hx == 0 , "No", "Yes")))

## Figure A.6. Labbe Plot -------------------------------------------------------
lab.xy = list("Control Group Estimate", "Treatment Group Estimate")
plot_labbe(dat = dat,
           covari.sel = c(4, 5, 6, 7),
           trt.sel = 3,
           resp.sel = c(1, 2),
           outcome.type = "survival",
           effect = "RMST",
           lab.xy = lab.xy,
           size.shape = 0.2,
           adj.ann.subgrp = 1 / 30,
           font.size = c(1, 1, 0.85, 1),
           time = 50, show.ci = FALSE, legend.position = "outside")
```

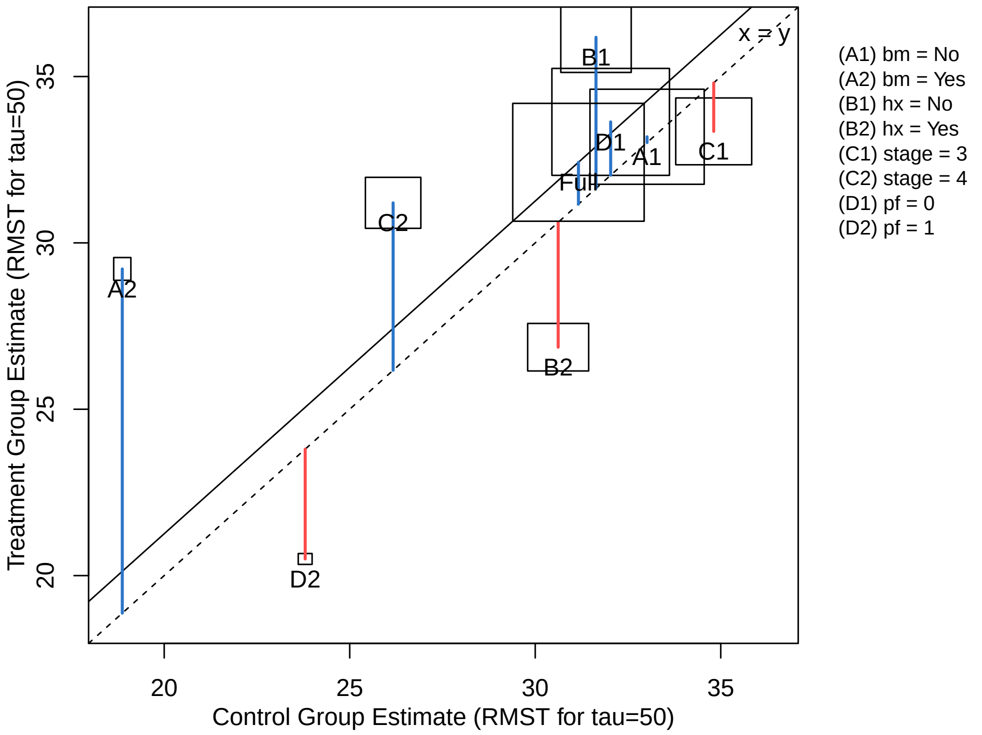

Figure S2.13: L'Abbé plot for subgroups defined by performance (pf), stage, history of cardiovascular events (hx) and existence of bone metastasis (bm). Effect sizes are given in terms of the difference in restricted mean survival time (RMST).

3.7 Chord diagram

```
dat =  prca
levels(dat$age_group)    = c("Young", "Middle-aged", "Old")
levels(dat$weight_group) = c("Low", "Mid", "High")
dat = dat %>%
  rename(Age = age_group,
         Weight = weight_group)

set.seed(55643)
# Figure A.7. Chord diagram ----------------------------------------------------
plot_circle(dat,
            covari.sel = c(14, 15),
            trt.sel = 3,
            resp.sel = c(1, 2),
            outcome.type = "survival",
            range.v = NULL, adj.ann.subgrp = 4,
            range.strip = c(-3, 3),
            n.brk = 31,
            n.brk.axis = 7,
            font.size = c(1, 1, 1, 1, 1),
            title = NULL, lab.xy = NULL,
            strip = "Treatment effect size (log hazard ratio)",
            effect = "HR",
            equal.width = FALSE,
            show.KM = FALSE,
            show.effect = TRUE,
            conf.int = FALSE, palette = "hcl")
#> Overall Treatment effect is: -0.1774, with confidence interval: (-0.3912;0.0365)
```

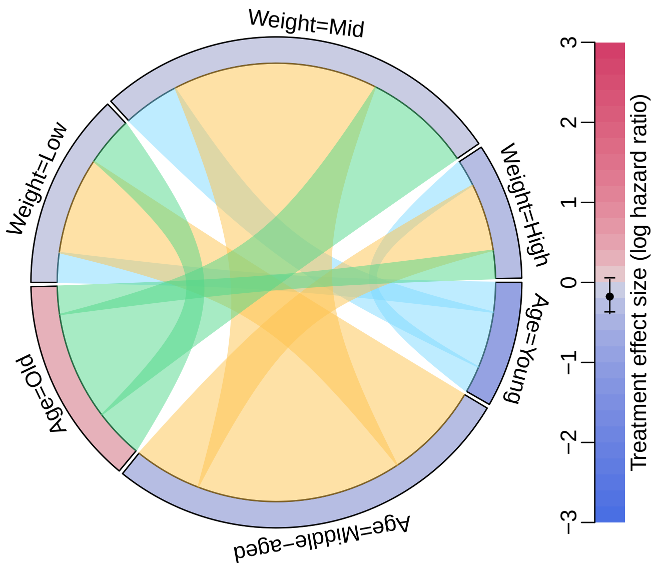

Figure S2.14: Chord diagram for the subgroups formed by age and weight. The colours along the circle represent the treatment effect in terms of the log-hazard ratio. The ribbons that link the subgroups represent their overlap.

3.8 Nightingale Rose

```
dat = prca
vars = data.frame(variable = names(dat), index = 1:length(names(dat)))
levels(dat$age_group) = c("Young", "Middle-aged", "Old")
levels(dat$weight_group) = c("Low", "Mid", "High")
names(dat)[c(14, 15)] = c("Age", "Weight")
strip.title = "Treatment effect size (log hazard ratio)"
## Figure A.8. Coxcomb plot
plot_nightingale_effect(dat,
                        covari.sel = c(14, 15),
                        trt.sel = 3,
                        resp.sel = c(1, 2),
                        outcome.type = "survival",
                        seq_by = 50,
                        range.strip = c(-3, 3),
                        n.brk = 31,
                        n.brk.axis =  7,
                        title = "Total sample size = 475",
                        strip = strip.title, effect = "HR",
                        show.overall = TRUE, palette = "hcl")
#> The minimum of treatment effect sizes is -2.426052
#> The maximum of treatment effect sizes is 0.3349348
```

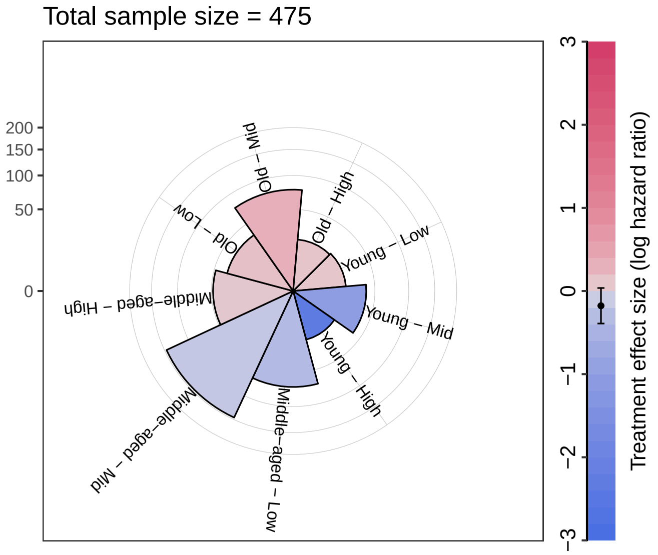

Figure S2.15: Nightingale coxcombs plot for subgroups defined by age and weight. The colours represent the treatment effect in terms of the log-hazard ratio. The radius of the sectors are proportional to the square root of the sample sizes in the subgroups.

4 Graphics in the supplementary material

4.1 Alternative Galbraith plot

```
dat = prca
dat = dat %>%
  mutate(bm = factor(ifelse(bm == 0 , "No", "Yes")),
         hx = factor(ifelse(hx == 0 , "No", "Yes")))
## Figure S1.1 Galbraith plot ----------------------------------------------------
p = ggplot_radial2(dat,
              covari.sel = c(4, 5, 6, 7),
              trt.sel = 3,
              resp.sel = c(1, 2),
              outcome.type = "survival",
              range.v = c(-11, 9),
              font.size = 4,
              lab.xy = "default",
              ticks.length = 0.05)
p +
  ggplot2::theme(text = ggplot2::element_text(size = 14))
```

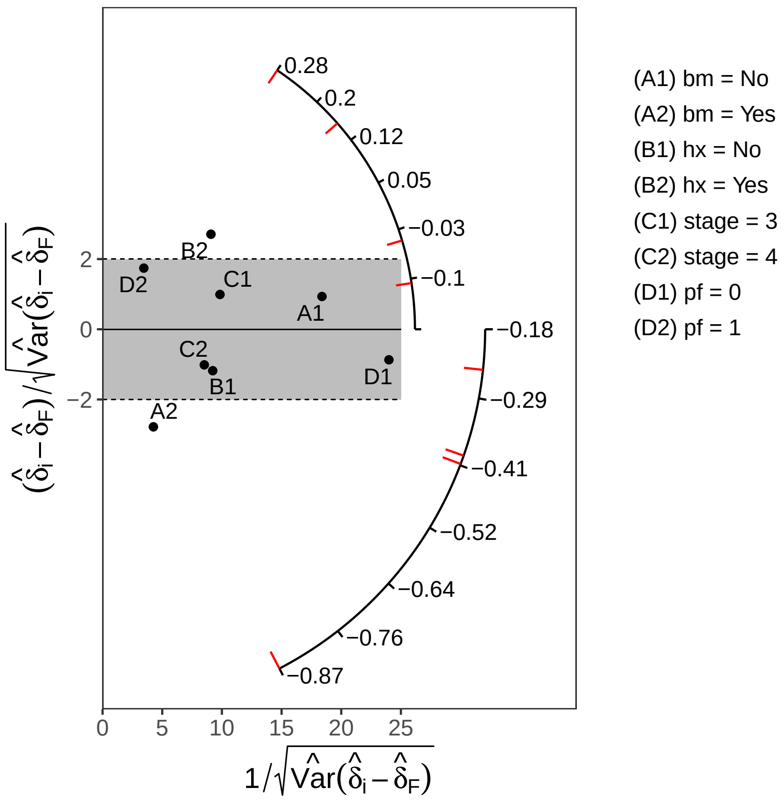

Figure S2.16: Modified Galbraith plot across subgroups defined by stage, history of cardiovascular events (hx) and existence of bone metastasis (bm)

4.2 Alternative STEPP plot with improper axis

```
dat = prca

## Figure S1.2 STEPP Plot -------------------------------------------------------------
lab.y.title = paste("Treatment effect size (log-hazard ratio)");
setup.ss = c(30, 40)
sub.title = paste0("(Subgroup sample sizes are set to ", setup.ss[2],
                   "; overlap of ", setup.ss[1], ")")
plot_stepp(dat,
           covari.sel = 8,
           trt.sel = 3,
           resp.sel = c(1, 2),
           outcome.type = "survival",
           setup.ss = setup.ss,
           alpha = 0.05,
           title = NULL,
           lab.y = lab.y.title,
           subtitle = sub.title)
#> The subgroup sample sizes are actually 46 44 43 44 42 56 48 48 61 53 75 64 111 83 47 87 83 79 74 61 87
```

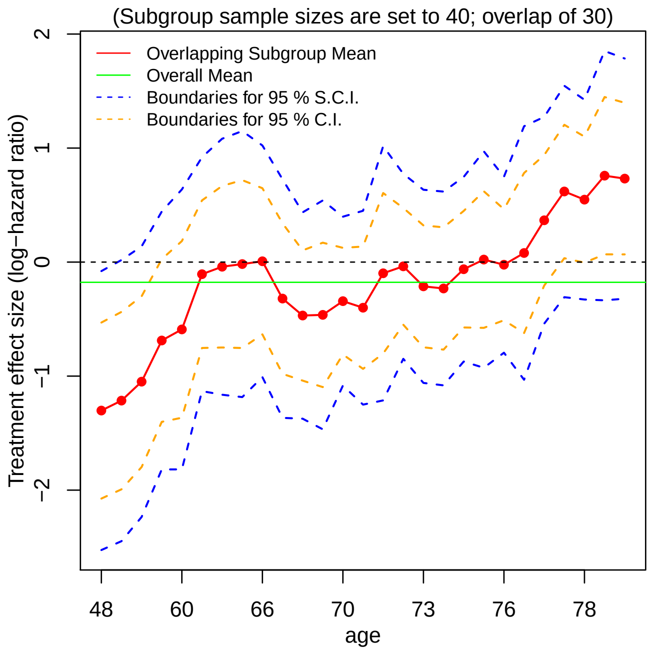

Figure S2.17: STEPP plot of overlapping subgroups defined by age. Each subgroup has a sample size of around $N_{11} = 40$ and is controlled to have about $N_{11} = 30$ subjects overlapping with the neighbouring subgroups.

4.3 Graphical approaches with an indirect comparison of treatment effects

4.3.1 Mosaic Plot

```
dat = prca
dat = dat %>%
  mutate(bm = factor(ifelse(bm == 0 , "No", "Yes")),
         hx = factor(ifelse(hx == 0 , "No", "Yes")),
         Treatment = factor(ifelse(rx == 0 , "Ctrl", "Exp")),
         Survival = factor(ifelse(survtime > 24 , "Yes", "No"),
                           levels = c("Yes", "No")))
levels(dat$age_group) = c("Young", "Middle-aged", "Old")
levels(dat$weight_group)  = c("Low", "Mid", "High")
vars = data.frame(variable = names(dat), index = 1:length(names(dat)))
# Change variable names
dat = dat %>%
  rename(`Bone Metastasis` = bm,
         `Performance rating` = pf,
         `History of cardiovascular events` = hx,
         `2-year survival` = Survival,
         Weight = weight_group,
         Age = age_group)

# Figure S1.3. Mosaic plot -------------------------------------------------------
plot_mosaic(dat,
            covari.sel = c(14, 16, 17),
            trt.sel = 3,
            resp.sel = c(1, 2),
            outcome.type =  "survival",
            range.v = NULL, adj.ann.subgrp = 4,
            range.strip = c(-3, 3),
            n.brk = 7,
            font.size = c(12, 12, 12, 12, 0.7),
```

```
             title = NULL, lab.xy = NULL, sep. = 0.03,
             strip = "Treatment effect size",
             effect = "HR", show.effect = FALSE)
```

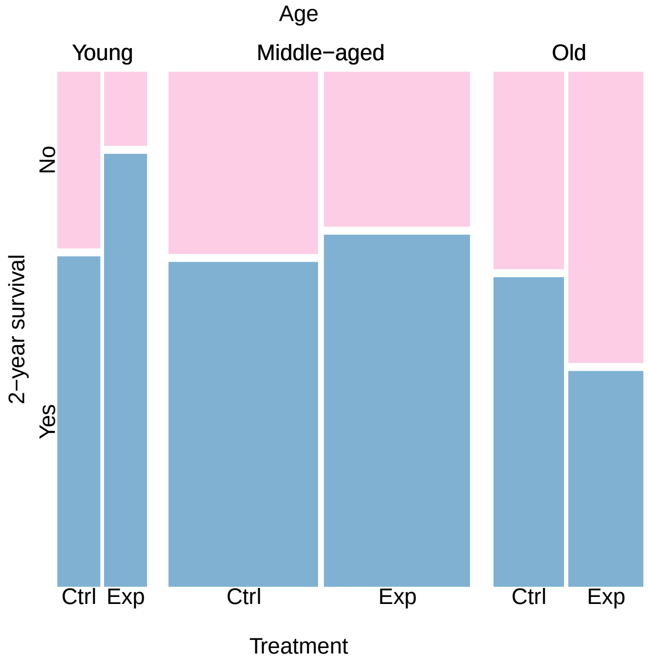

Figure S2.18: Mosaic plot displaying 2-year survival by treatment arm for the subgroups formed by age categories.

4.3.2 Nightingale Rose

```
dat = prca
levels(dat$age_group) = c("Young", "Middle-aged", "Old")
levels(dat$weight_group)  = c("Low", "Mid", "High")
comb_levels = c("Young - Low", "Young - Mid", "Young - High",
                "Middle-aged - Low", "Middle-aged - Mid", "Middle-aged - High",
                "Old - Low", "Old - Mid", "Old - High")
dat = dat %>%
  mutate(AgeWeight = factor(sprintf("%s - %s", age_group, weight_group),
                            levels = comb_levels))  %>%
  mutate(survival = factor(ifelse(survtime > 24 , "Yes", "No"),
                           levels = c("No", "Yes"))) %>%
  mutate(rx = factor(rx, labels = c("Control", "Treatment")))


## Figure S1.4. Coxcomb Plot ---------------------------------------------------
plot_nightingale(dat = dat,
                 covari.sel = 16,
                 resp.sel = 17,
                 strip = "2-year survival")
```

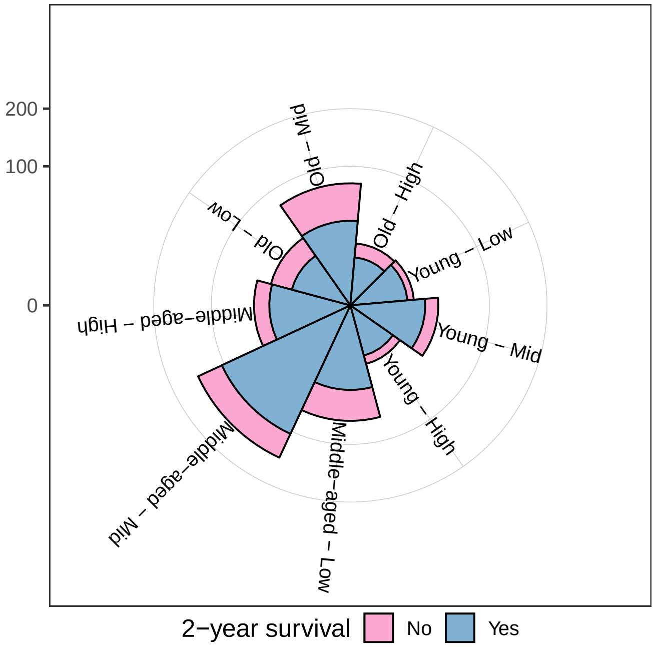

Figure S2.19: Nightingale coxcombs plot for subgroups defined by age and weight with 2- year survival rate. The radius of the sectors are proportional to the square root of the sample sizes in the subgroups

```
## Figure S1.5. Coxcomb Plot 2 separate arms -----------------------------------
plot_nightingale(dat = dat, trt.sel = 3,
                 covari.sel = 16,
                 resp.sel = 17,
                 seq_by = 50,
                 strip = "2-year survival")
```

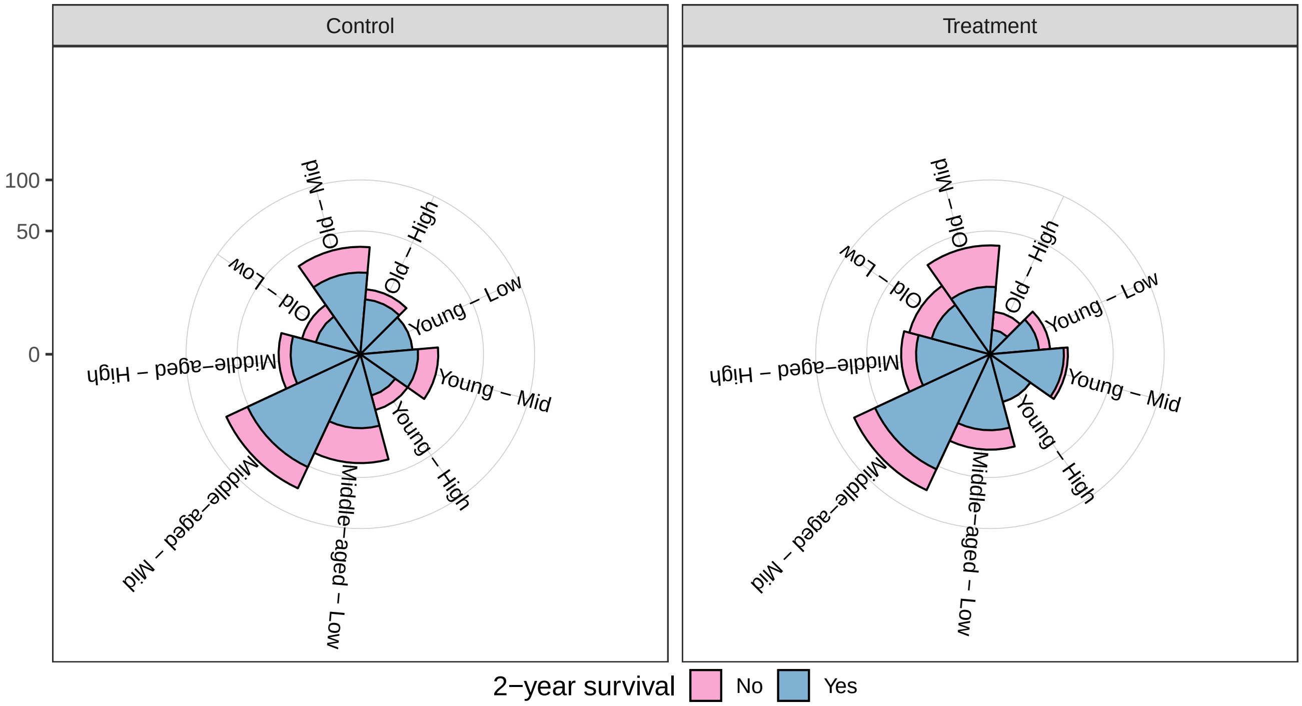

Figure S2.20: Nightingale coxcombs plot for subgroups defined by age and weight with 2-year survival rate and separated by treatment arm. The radius of the sectors are proportional to the square root of the sample sizes in the subgroups

4.3.3 Alluvial Plot

```
dat = prca
dat = dat %>%
  mutate(survival = factor(ifelse(survtime > 24 , "Yes", "No"), levels = c("No", "Yes")),
         trt = rx)
alldat = dat %>%
  dplyr::select(trt, bm, hx, pf, survival) %>%
  dplyr::group_by(trt, bm, hx, pf, survival) %>%
  dplyr::summarise(Freq = n())
alldat = alldat %>%
  ungroup() %>%
  mutate(trt = ifelse(trt == 0 , "Control", "Treatment"),
         bm = ifelse(bm == 0 , "No", "Yes"),
         hx = ifelse(hx == 0 , "No", "Yes"))

# Figure S1.6. Alluvial diagram by survival --------------------------------------
plot_alluvial(alldat[, c(5, 1, 3, 2, 4)], freq = alldat$Freq,
              xw = 0.2, cw = 0.12, cex = 1,
              alpha  = 0.8,
              col = ifelse(alldat$survival  == "Yes",
                           ifelse(alldat$trt  == "Treatment", "#80b1d3", "#d5e2eb"),
                           ifelse(alldat$trt  == "Treatment", "#faa8d2", "#fbe0ee")),
              layer = alldat$trt  == 1, rotate = 90, las = 2, bottom.mar = 5)


# -------------------------------------------------------------------------------
dat = prca
dat$trt = dat$rx
alldat = dat %>%
  dplyr::select(trt, bm, hx, pf) %>%
  dplyr::group_by(trt, bm, hx, pf) %>%
  dplyr::summarise(Freq = n())
alldat = alldat %>%
  ungroup() %>%
  mutate(trt = ifelse(trt == 0 , "Control", "Treatment"),
         bm = ifelse(bm == 0 , "No", "Yes"),
         hx = ifelse(hx == 0 , "No", "Yes"))

# Figure S1.7. Alluvial diagram by treatment arms --------------------------------
plot_alluvial(alldat[, c(1, 3, 2, 4)], freq = alldat$Freq,
              xw = 0.2, cw = 0.12, cex = 1,
              alpha  = 0.8,
              col=ifelse(alldat$trt == "Treatment", "#1f78b4", "#a6cee3"),
              layer = alldat$trt  == 1, rotate = 90)
```

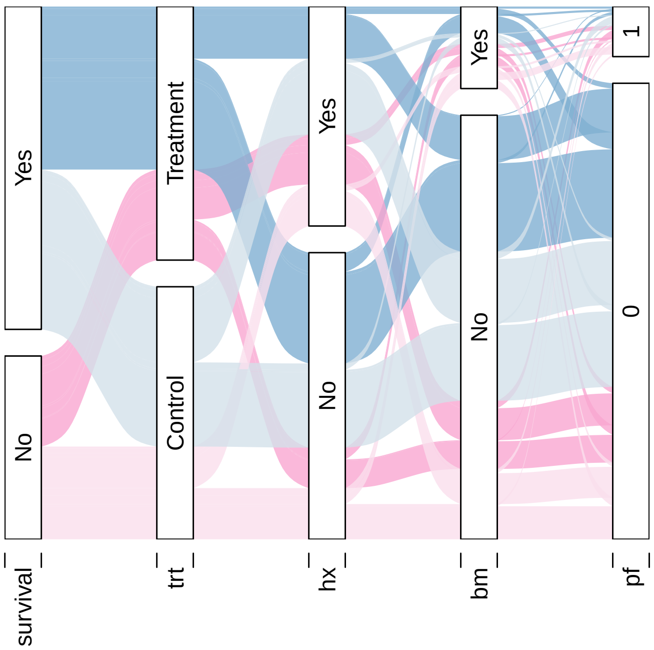

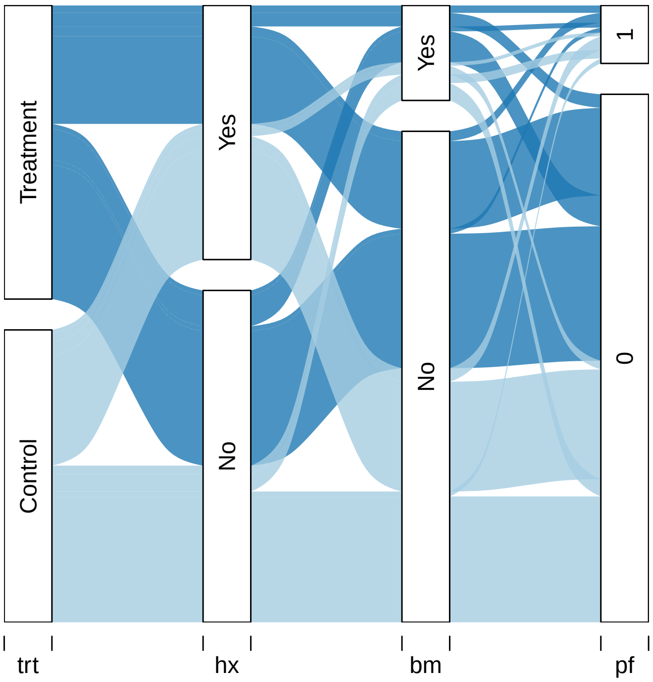

Figure S2.21: Alluvial diagram displaying the distribution of patients across the subgroups defined by history of cardiovascular events (hx), existence of bone metastasis (bm) and performance rating (pf). The dark bands correspond to patients that were randomised to treatment while lighter ones to patients in control. Blue coloured bands represent patients that had survived for at least 2 years, while pink ones represent those who did not. The width of the bands is proportional to the sizes of the subgroups.

Figure S2.22: Alluvial diagram displaying the distribution of patients across the subgroups defined by history of cardiovascular events (hx), existence of bone metastasis (bm) and performance rating (pf). The dark blue bands correspond to patients that were ran- domised to treatment while light blue ones to patients in control. The width of the bands is proportional to the sizes of the subgroups.

4.4 Graphical approaches for subgroup composition

4.4.1 Pairwise overlap

```
dat = prca


## Figure S1.8. Overlap plots  -------------------------------------------------

## Figure S1.8.a Overlap plot ----------------------------------------------------
plot_overlap(dat = dat,
             covari.sel = c(6, 5, 4, 7),
             para = c(0.1, 0.5, 1),
             font.size = c(1.2, 1.2, 0.8),
             title = NULL)
## Figure S1.8.b Overlap alternative plot ----------------------------------------
plot_overlap_alternative(dat = dat,
                         covari.sel = c(6, 5, 4, 7),
                         mode = 1,
                         para = c(0, 0.6, 1),
                         font.size = c(1.2, 1.2, 0.8),
                         title = NULL)
## Figure S1.8.c Network plot ----------------------------------------------------
plot_network(dat = dat,
```

```
              covari.sel = c(6, 5, 4, 7),
              para = c(0.1, 0.5, 1),
              font.size = c(1.2, 1.2, 0.8),
              title = NULL)
## Figure S1.8.d Matrix Overlap plot ------------------------------------------
plot_matrix_overlap(dat,
                    covari.sel = c(6, 5, 4, 7),
                    mode = 1,
                    font.size = c(1.5, 1.25, 0.8),
                    title = NULL)
## Figure S1.8.e dissimilarity plot ----------------------------------------------
plot_dissimilarity(dat = dat,
                   covari.sel = c(4, 5, 6),
                   mode = 3,
                   range.ds = c(0, 1),
                   font.size = c(1, 0.9, 1, 0.7),
                   title = NULL,
                   lab.x = "Dissimilarity distance")
## Figure S1.8.f dissimilarity alternative plot ----------------------------------
plot_dissimilarity_alternative(dat = dat,
                               covari.sel = c(4, 5, 6),
                               mode = 2,
                               range.ds = c(0, 1),
                               font.size = c(1, 1, 0.7),
                               title = NULL,
                               lab.y = "Similarity distance")
```

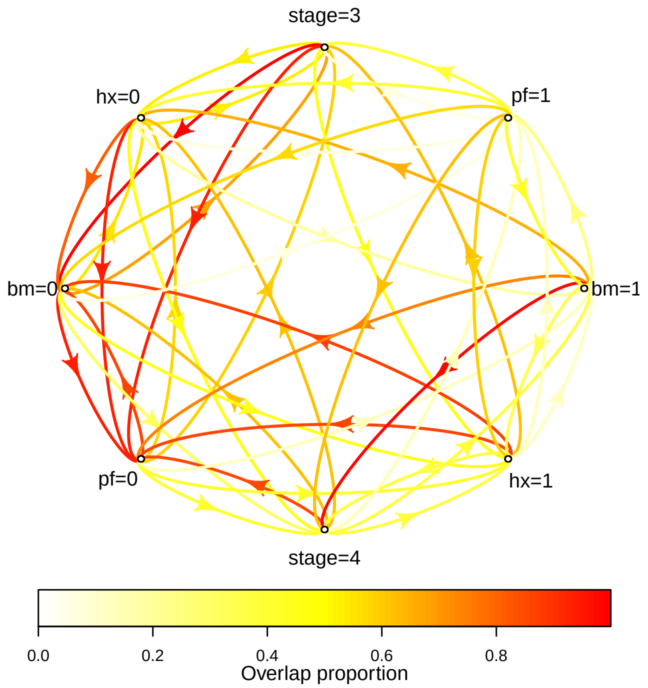

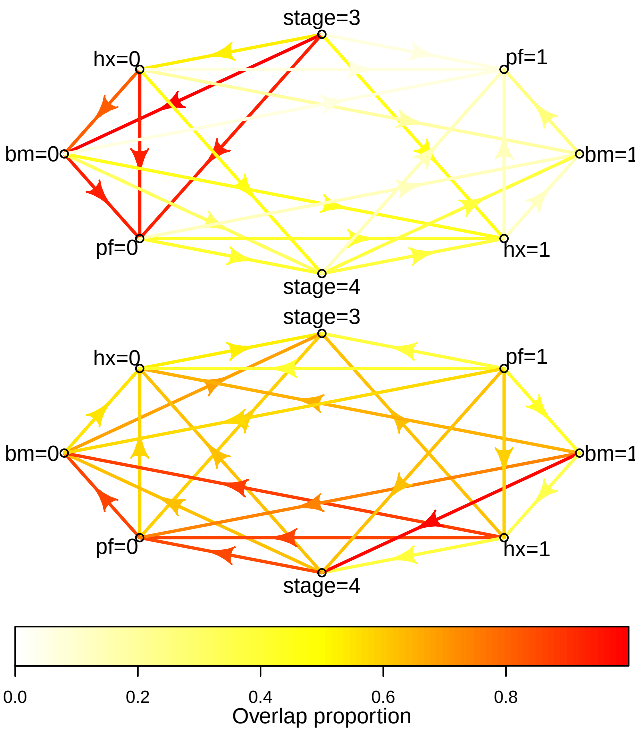

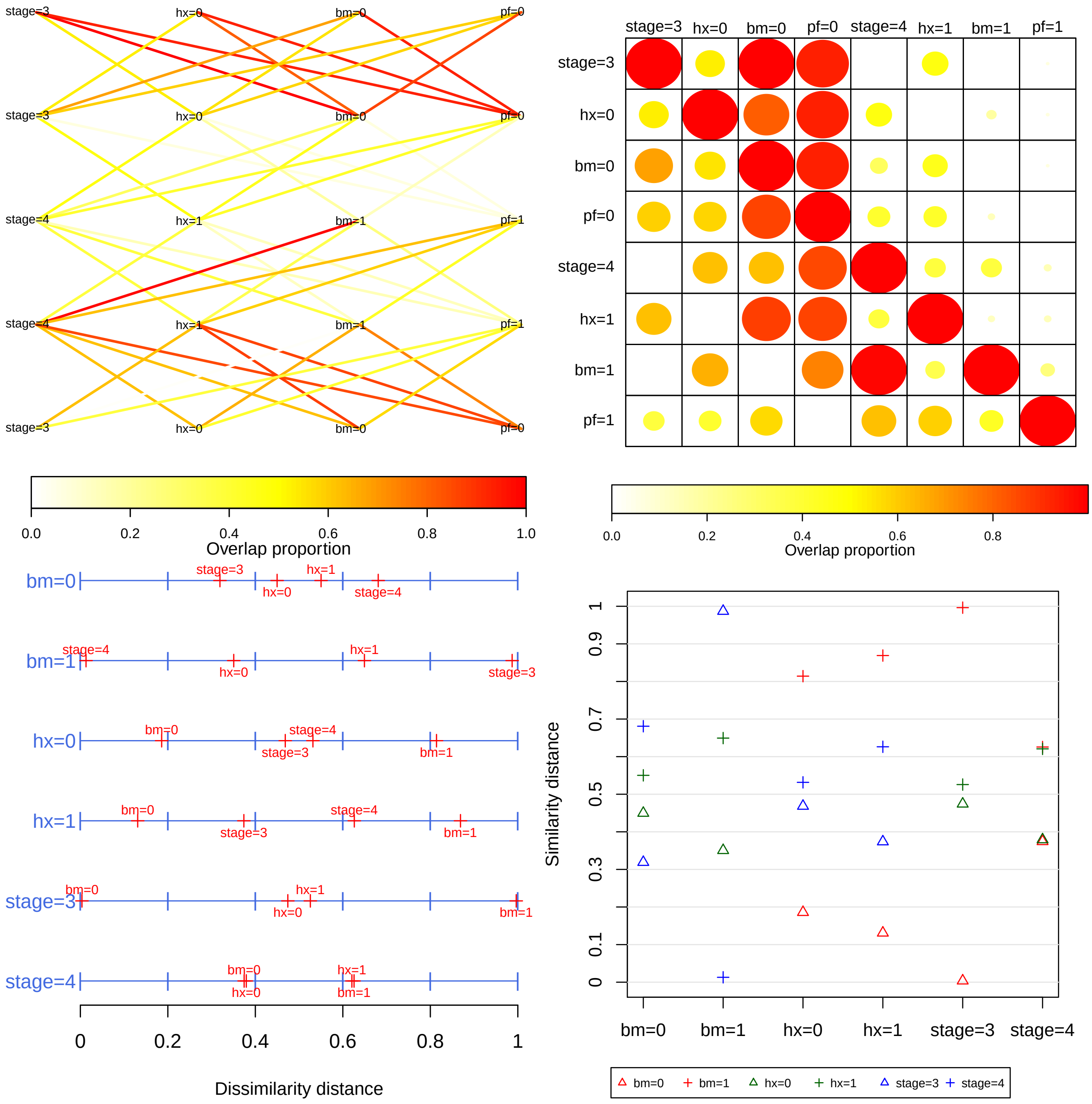

Figure S2.23: Plots for subgroup information about pairwise overlap proportions or dissimilarity measure.

4.4.2 Chord diagrams in matrix layout

```
dat = prca
set.seed(12)
## Figure S1.9. Overlap plots ------------------------------------------------
plot_circle2(dat,
             covari.sel = c(4, 5, 6, 7),
             trt.sel = 3,
             resp.sel = c(1, 2),
             outcome.type = "survival",
             range.v = NULL,
             adj.ann.subgrp = 4,
             range.strip = c(-3, 3),
```

```
              n.brk = 31,
              n.brk.axis = 7,
              font.size = c(1, 1, 1.75, 0.85, 1),
              title = NULL, lab.xy = NULL,
              strip = "Treatment effect size (log hazard ratio)",
              effect = "HR",
              equal.width = FALSE,
              show.KM = FALSE,
              show.effect = TRUE,
              conf.int = FALSE, palette = "hcl")
```

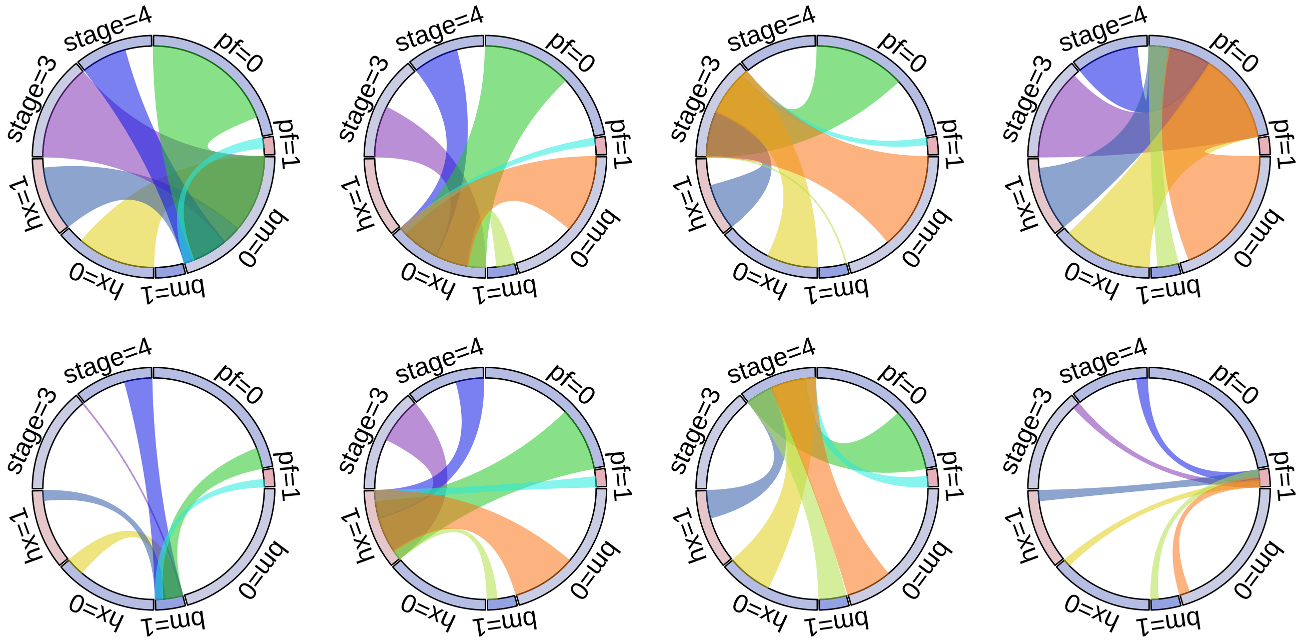

Figure S2.24: Chord diagrams displaying the subgroups formed by the categorized age and weight covariates. The width of each section is proportional to the sample size in the corresponding subgroup. Each circle displays the relative overlap of one subgroup with the others.

```
dat = prca
set.seed(12)
## Figure S1.10. Overlap plots ------------------------------------------------
plot_circle2(dat,
              covari.sel = c(4, 5, 6, 7),
              trt.sel = 3,
              resp.sel = c(1, 2),
              outcome.type = "survival",
              range.v = NULL, adj.ann.subgrp = 4,
              range.strip = c(-3, 3),
              n.brk = 31,
              n.brk.axis = 7,
              font.size = c(1, 1, 1.75, 0.85, 1),
              title = NULL, lab.xy = NULL,
              strip = "Treatment effect size (log hazard ratio)",
              effect = "HR",
              equal.width = TRUE,
              show.KM = FALSE,
              show.effect = TRUE,
              conf.int = FALSE, palette = "hcl")
```

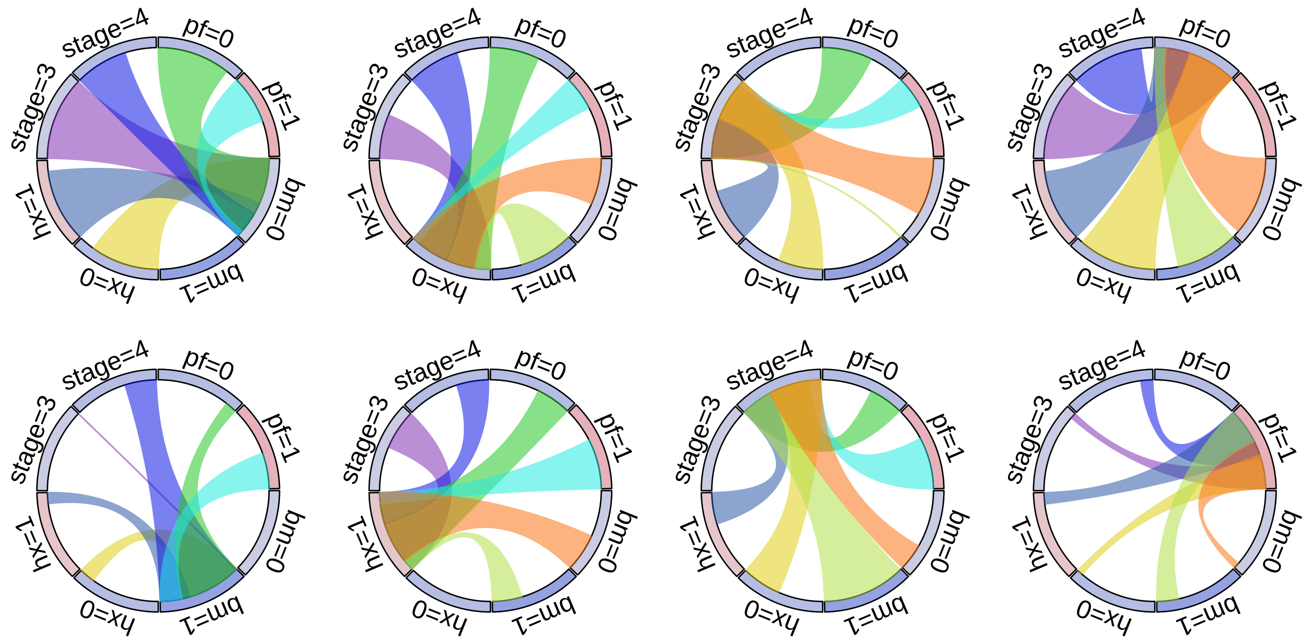

Figure S2.25: Chord diagrams displaying the subgroups formed by the categorised age and weight covariates. Sample sizes are not depicted in this version as the widths are the same for each section. Each circle displays the relative overlap of one subgroup with the others.

```
dat = prca
## Figure S1.11. Overlap plots ---------------------------------------------------
plot_overlap2(dat = dat,
              covari.sel = c(6, 5, 4, 7),
              para = c(0.05, 0.75, 1),
              font.size = c(1.2, 1.2, 0.8),
              title = NULL)
```

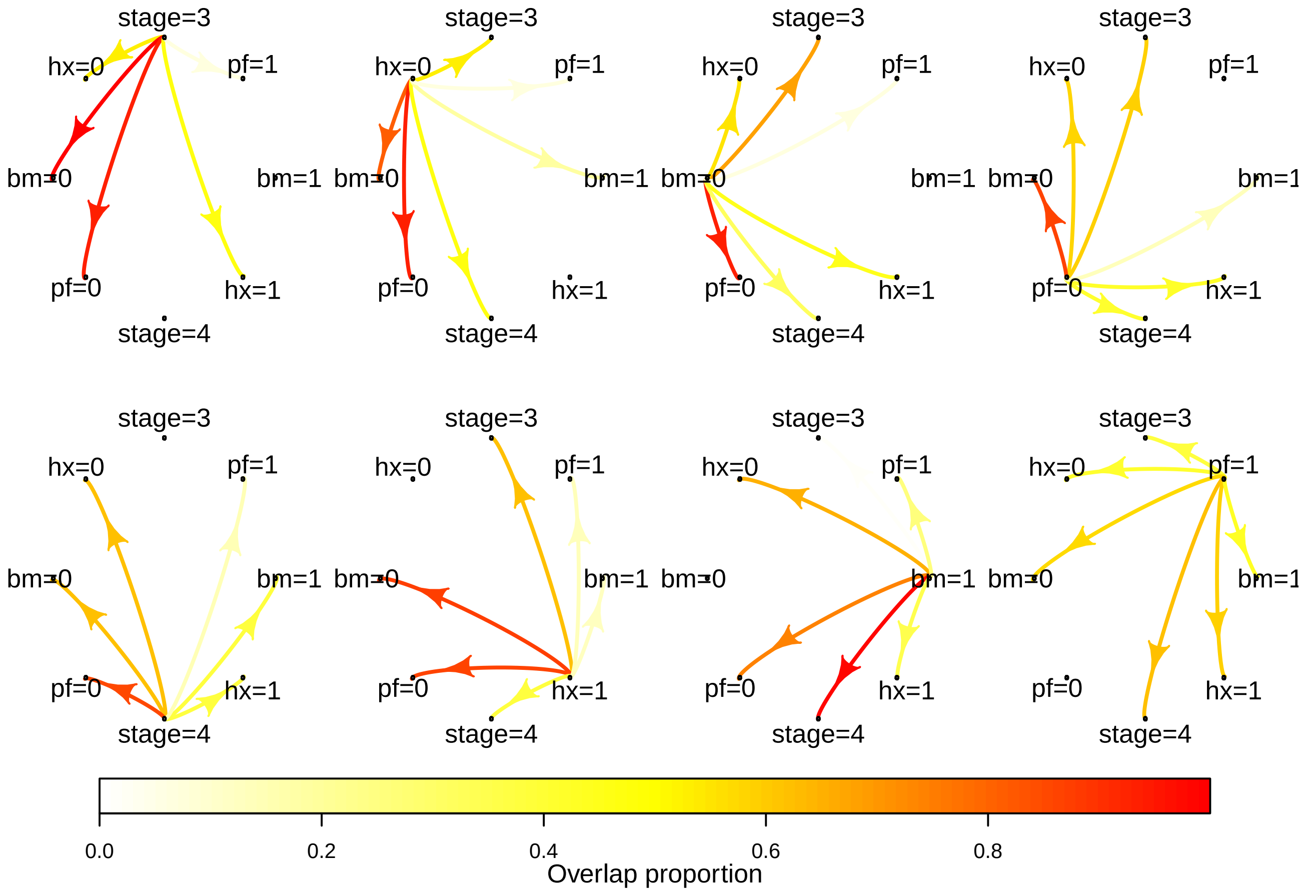

Figure S2.26: Line plots with unidirectional arrowed lines for relative overlap proportions for pairwise subgroups. Each subplot contains the overlap of one subgroup with all the others.

5 R Session Info

```
sessionInfo()
#> R version 3.5.1 (2018-07-02)
#> Platform: x86_64-apple-darwin15.6.0 (64-bit)
#> Running under: macOS  10.14.6
#>
#> Matrix products: default
#> BLAS: /Library/Frameworks/R.framework/Versions/3.5/Resources/lib/libRblas.0.dylib
#> LAPACK: /Library/Frameworks/R.framework/Versions/3.5/Resources/lib/libRlapack.dylib
#>
#> locale:
#> [1] en_US.UTF-8/en_US.UTF-8/en_US.UTF-8/C/en_US.UTF-8/en_US.UTF-8
#>
#> attached base packages:
#> [1] stats     graphics  grDevices utils     datasets  methods   base
#>
#> other attached packages:
#> [1] UpSetR_1.3.3     bindrcpp_0.2.2   dplyr_0.7.7      SubgrPlots_0.1.3
#>
#> loaded via a namespace (and not attached):
#>  [1] circlize_0.4.5      shape_1.4.4         diagram_1.6.4
#>  [4] tidyselect_0.2.5    xfun_0.6            purrr_0.2.5
#>  [7] reshape2_1.4.3      splines_3.5.1       lattice_0.20-35
```

```
#> [10] colorspace_1.3-2     htmltools_0.3.6      yaml_2.2.0
#> [13] survival_2.42-3      rlang_0.3.0.1        pillar_1.3.0
#> [16] glue_1.3.1           sp_1.3-1             lambda.r_1.2.3
#> [19] bindr_0.1.1          plyr_1.8.4           stringr_1.3.1
#> [22] rgeos_0.4-2          munsell_0.5.0        gtable_0.2.0
#> [25] futile.logger_1.4.3  GlobalOptions_0.1.0  codetools_0.2-15
#> [28] evaluate_0.13        labeling_0.3         knitr_1.22
#> [31] Rcpp_0.12.19         scales_1.0.0         formatR_1.5
#> [34] gridExtra_2.3        ggplot2_3.2.0        digest_0.6.18
#> [37] stringi_1.2.4        ggrepel_0.8.0        survRM2_1.0-2
#> [40] polyclip_1.9-1       grid_3.5.1           tools_3.5.1
#> [43] magrittr_1.5         lazyeval_0.2.1       tibble_1.4.2
#> [46] futile.options_1.0.1 crayon_1.3.4         pkgconfig_2.0.2
#> [49] Matrix_1.2-14        assertthat_0.2.0     rmarkdown_1.13
#> [52] R6_2.3.0             VennDiagram_1.6.20   compiler_3.5.1
```
